# Transition in the ages at key reproductive events and its determinants in India: evidence from NFHS 1992-93 to 2019-21

**DOI:** 10.1186/s12905-023-02271-w

**Published:** 2023-03-29

**Authors:** Mayank Singh, Chander Shekhar, Jagriti Gupta

**Affiliations:** Department of fertility & Social Demography, International Institute for Population Sciences (IIPS), Mumbai, 400088 India

**Keywords:** Reproductive events, Women’s Health, First Cohabitation, First sex, First Birth, MCA, MDA, India

## Abstract

**Introduction:**

Reproductive health events have changed fertility and family planning needs, depicting the changing life patterns of women and the population to which they belong. Understanding the pattern at which these events occur helps in understanding the fertility pattern, family formation and the idea about health essential needs for women. This paper attempts to see the variation in reproductive events (first cohabitation, first sex and first birth) over three decades and also to see potential contributing factors among the reproductive age group of women using secondary data from Data Source: All rounds of the National Family Health Survey (1992-93 to 2019–2021) have been utilized.

**Methods and Results:**

Cox Proportional Hazard Model illustrates that all regions have initiated their first birth later than women who belong to the east region similar pattern has been obtained for first cohabitation and first sex except for the central region. Multiple Classification Analysis (MCA) depicts the increasing pattern in the predicted mean age at first cohabitation, sex and birth for all demographic characteristics; the highest increment was found in SC women, Uneducated women and Muslim women. Kaplan Meier Curve demonstrates that women with no education, primary or secondary education are shifting towards higher educated women. Most importantly, the results of the multivariate decomposition analysis (MDA) revealed that education played the largest contribution among the compositional factors in the overall increase in mean ages at key reproductive events.

**Conclusions:**

Though reproductive health has long been essential in women’s lives, they are still very confined to specific domains. Over time the government has formulated several proper legislative measures relating to various domains of reproductive events. However, given that the large size and heterogeneity in social and cultural norms result in changing ideas and choices regarding the initiation of reproductive events, national policy formulation needs to be improved or amended.

**Supplementary Information:**

The online version contains supplementary material available at 10.1186/s12905-023-02271-w.

## Introduction

Women’s overall health, well-being and its consequences represents the reproductive health scenario of any nation. According to WHO, reproductive health is defined as “a state of complete, physical, mental and social well-being and not merely the absence of disease or infirmity, in all matters relating to the reproductive system and to its function and processes. Reproductive health implies that people are able to have a satisfying and safe sex life and they have the capability to reproduce and the freedom to decide it when and often to do so” [1]. The reproductive health status of any women starts from event of menarche and ends at the event of menopause whereas the consequences of occurrence of these events continues throughout the life [2]. Many events between these two events such as age at marriage, age at cohabitation, age at first intercourse, age at first pregnancy, age at first birth, all are indicative of reproductive health status of a women for current life and later life as well [2, 3]. There is existence of variation in the occurrence of all these reproductive events among the countries, within the countries or within any population [4]. Within population it also varies by residence, caste, religion and socio-economic status [5, 6]. All reproductive events are linked to each other and have their own implications and effects on women’s health, society, and the whole population [7]. For a glance, younger age at marriage results in long period of exposure to child bearing [8] but this phenomenon varies by country to country, in many developed countries age at first marriage does not affects or does not result in longer period of childbearing whereas in countries like India marriage has been the most important events of reproductive health [9]. India’s population growth and development strongly depends on age at marriage residing there [10]. The reproductive health events lead to family formation and depicts the fertility of any country. Individual lives vary with the order and timing of events, reflects the social and environmental surrounding subsequently there is existence of generation gap in the occurrence of these reproductive events. This gap also varies with the demographic trends, socio-economic status as well as change in cultural and social norms [11].

There has been an occurrence of transformation and shifting in the union formation of people throughout the world, now people are marrying at the highest age on record and young adults started to believe in cohabitation [12, 13]. A study done by Manning in the USA found that the majority of young adults have spent some in a cohabiting union [13], about two-fifth of women married in the early 1980s cohabited prior to entering a marriage that is preceded by cohabitation [14]. A study done by Bumpass, Sweet and Cherlin in 1991 found that delay in marriage entry evident in the 1970 and 1980s offset by subsequent increase in cohabitation formation was stable, i.e., cohabitation was unchanged over time, whereas the type of the first union formed shifted from marriage to cohabitation [15]. In literature, very little attention has been given to age at first cohabitation since very few data is available to document these shifts.

Among various reproductive events, the one crucial event is sexual intercourse and is generally initiated during adolescence [2]. Early initiation of sexual intercourse may lead to an increase in the risk of sexually transmitted diseases and premature or premarital pregnancy [16]. Early initiation of sexual intercourse also causes biological predisposition of the immature cervix to infection, which results in the increased likelihood of engaging in riskier sexual behavior among persons who initiated sexual intercourse at younger ages i.e. age at first sex may represent a life course transition that increases the likelihood of a longitudinal pattern of risky sexual activity [17]. Early initiation of sexual intercourse may lead to many negative consequences and affect sexual functioning and relationship skills [18]. Therefore, age at first sex is an important indicator over the life cycle of a person, especially of a woman who is facing various social and cultural norms on a daily basis, particularly in countries like India.

Early cohabitation and early birth resultant of early marriage is common practice in many parts of the developing world, with the significant number of women being cohabited or having early first birth before the age of eighteen [19]. Cohabitation is a kind of formation of union, this formation of union can be after marriage or before marriage. Every individual has the right to form a union. However, this union does not mean only the union of marriage it can be in a form of cohabitation which means “companionship in each form of word which is either sexual, mental, physical or emotional. An important aspect of cohabitation is that it’s an individual’s choice as what kind of partner he/she wants or what kind of relationship he/she wants with that partner [20]. In Indian scenario cohabitation occurs only after marriage, here marriage is traditionally two stage phenomena where marriage and cohabitation separated by certain time interval, after marriage when partner started to live together then form a cohabitation [9].

There are various studies that showed a negative relationship between early cohabitation, early marriage or early age at first birth and health and education outcomes of women and their children [21]. There are various reasons why age at cohabitation, age at sex and age at first birth matter. First early cohabitation or early age at first birth implies by early age at marriage leads to dropping out of the school of women [22]. Field and Ambrus found in 2008 that marriage delayed results in 0.22 more years of schooling in the developing world; henceforth, delayed marriage results delay in the age of cohabitation and age of first birth [23]; secondly, women who marry early might marry into worse household than average one therefore early age of cohabitation, age of sex or age of first birth impact on household decision making, women’s fertility and freedom of mobility [24], overall age at which these events occur important for the upcoming generation, the implications are not only affected by educational attainment of women or women’s husband but the age at which these events occur matter by itself [25]. In a study, it was found that in India, 50% of women married before the age of eighteen, which is the minimum legal age of marriage, whereas in some states of India incentives were also provided in spite of that there was no such significant difference found hence there is the rationale of direct policy intervention to increase the age at marriage which will imply an increase in age of cohabitation and age of first birth [10].

The timing and order of these reproductive events are compound and vacillating, subsequetly affecting individual lives. Ordering of occurrence of these events helps in understanding the life process and health as well as social status of that particular individual and similarly helps in comparing different social groups or subgroups at various stages [3]. All these reproductive events are directly related to infant and maternal health; therefore, knowledge of the occurrence of these events at typical ages is valuable and observing differences within the population or subpopulation helps to identify who faces a higher risk of certain problems or who has a lack of specific services [26]. Timing of occurrence of these events and stability of family formation concur with education, income and exposure to specific facilities lead to anticipate root and ramifications of economic and health inequality [27, 28]. In this paper, our study aims to see the variation in these events (first cohabitation, first sex and first birth) over the period of three decades and also to identify the potential factors contributing to changing age at reproductive events.

### Data and methods

#### Data source

Data were drawn from all five rounds of demographic and health survey conducted during 1999–1993 (NFHS-1), 1998–1999 (NFHS-2), 2005–2006 (NFHS-3), 2015–2016 (NFHS-4) and 2019-21 (NFHS-5) in India, popularly known as National Family Health Survey (NFHS). The key purpose of the survey is to provide a wide range of up-to-date and reliable information about health and demographic indicators such as maternal and child health, fertility, childhood mortality and morbidity, family planning, immunization, nutritional status, non-communicable disease, domestic violence and breastfeeding practices, etc. Furthermore, NFHS also contains information about the age at occurrence of reproductive events like age at first marriage, age at first cohabitation, age at first sex, age at first birth, etc. For the first three rounds of the survey (NFHS-I, NFHS-II and NFHS-III) uniform sampling procedure was adopted in all states. Two-stage sampling design was adopted for the rural area; PPS sampling procedure was used for the selection of villages after that random selection of households were taken place. Three stage sampling design was adopted for the urban areas, selection of wards with PPS sampling followed by random selection of Census enumeration block (CEB). Thereafter random selection of households was taken place. Additionally, in the last two rounds of the survey, two stage stratified sampling design was adopted. Selection of villages in rural areas (CEB in urban areas) was done by using PPS sampling and thereafter, random selection of households in both areas (rural and urban) was done by systematic sampling. The sampling frame used in the first round of the survey (NFHS-I) was 1981 census frame except for Assam, Delhi and Punjab (Census 1991 frame), whereas for all other rounds of the survey census conducted just before the survey was taken as a sampling frame for selection of sampling units [29–33].

### Variable description

#### Outcome variable

In this study, age at first cohabitation, age at first sex and age at first birth were taken as the outcome variable of interest. All those respondents who were cohabited were taken as the eligible respondent for age at first cohabitation. Similarly, respondents who were given the information about age at first sex and age at first birth were taken as the eligible respondent for age at first sex and first birth, respectively. During the survey following questions were asked to know about the ages of first cohabitation, first sex and first birth, (1) In what month and year did you start living with your husband? (2) Now I would like to ask about when you started living with your first husband. In what month and year was that? (3) How old were you when you first started living with him? (4) How old were you when you had sexual intercourse for the very first time? (5) In what month and year child was born? For all births of the respondent.

#### Predictor variable

Socio-demographic and other characteristics were included for the explanatory variable in this study. The operational definition and coding of the variables in this study are as follows: Place of Residence coded as 1 = Urban, 2 = Rural; Region is coded as 1 = East, 2 = West, 3 = North, 4 = South, 5 = Central, 6 = Northeast; Religion is coded as 1 = Hindu, 2 = Muslims, 3 = Christian, 4 = Others; Caste is coded as 1 = SC (Schedule Caste), 2 = ST (Schedule Tribe), 3 = OBC (Other Backward Caste), 4 = Others based on their responses on ethnicity; Respondent level of education is coded as 1 = No education, 2 = Primary, 3 = Secondary, 4 = Higher; Wealth index is coded as 1 = Poorest, 2 = Poorer, 3 = Middle, 4 = Richer, 5 = Richest; Mass media exposure is measure from frequency of reading newspaper/magazines, listening radio, watching television, go to the cinema hall or theatre to see a movie coded as 1 = No, 2 = Any; Household structure is coded as 1 = nuclear and 2 = Non-nuclear and lastly Prior relationship with husband is coded as 1 = No, 2 = Yes.

#### Statistical analysis

We have used the descriptive statistics, bivariate, Cox Proportional Hazard Model, Multiple Classification Analysis, Kaplan Meier Curve, Life table survival analysis, hierarchical clustered heat map, Multivariate decomposition analysis and geospatial mapping, to fulfil the objective of the study. Descriptive analysis was carried out to understand the distribution of background characteristics of variables for the study samples. Bivariate analysis was carried out to see distribution of age at first cohabitation, age at first sex and age at first birth by exact age over the current age of the respondent. We also calculated the Median age at first cohabitation, sex and birth among the women by current age in each round of the survey. Median age at events (first cohabitation, first sex, first birth) was calculated only for those current age groups which comprised of more than or equal to 50% of the respondents getting event done before reaching to the lower limit of that age group.

Geospatial mapping is used to show the state specific variation in predicted mean age at first cohabitation, sex and birth over the period of three decades. Additionally, failure life table estimates were picturized as hierarchal clustered heat map which is used to understand true probability of state specific non-occurrence of the above events. In this article, we have used the Kaplan Meier survival method to obtain probability of women still not cohabited, not having sex and not given birth by a particular age among overall eligible respondent by various sociodemographic characteristics.

Further to see the influence on the timing of event happening in a multivariable context, we had performed Cox Proportional hazard regression analysis. The Cox model is expressed by the hazard function denoted by h(t). Briefly, for the current study the hazard function can be defined as the risk of first cohabitation, sex and birth at time t. The equation for hazard model is given as follows:

$${\rm{h}}\left( {\rm{t}} \right){\rm{ }} = {\rm{ }}{{\rm{h}}_{\rm{0}}}\left( {\rm{t}} \right){\rm{ }} \times {\rm{ exp }}\left( {{{\rm{b}}_{\rm{1}}}{{\rm{x}}_{\rm{1}}}{\rm{ + }}{{\rm{b}}_{\rm{2}}}{{\rm{x}}_{\rm{2}}}{\rm{ + }}...{\rm{ + }}{{\rm{b}}_{\rm{p}}}{{\rm{x}}_{\rm{p}}}} \right)$$

In such a model, the outcome variable is the risk of hazard of experiencing the event of cohabitation, sex and birth, and the odds ratio for each independent variable represents the likelihood of experiencing the event for a particular group compared with the reference group. Furthermore, predicted mean age at reproductive events by various socio-economic and demographic characteristics was also calculated with the help of Multiple Classification Analysis. The advantage of the MCA convergence model was that we can estimate the values of reference category of the dependent variable which was not possible in cox proportional hazard model or simple linear regression analyses.

Additionally, multivariate decomposition analysis was performed to determine the change in mean age at key reproductive events and the factors that contributed to the change. The goal of the decomposition analysis was to determine the source of the shift in mean cohabitation, sex and age at first birth among reproductive-aged women over the last three decades (1992 to 2021). The multivariate decomposition analysis divides the overall increase in age at cohabitation, first sex and first birth over time into the increase caused by differences in women’s composition (endowment) between surveys and the increase caused by differences in the effect of the characteristics (coefficient) between surveys.

All the analysis was carried out using Stata statistical software version 16.1 (StataCorp, College Station, TX), ArcGIS & Origin Pro version 9.9.

## Results

Table 1 demonstrates the sample characteristics of first cohabitation, sex and birth among reproductive-aged women (15–49) years by background characteristics. It is observed that there has been a decline in the proportion of women of younger ages included in the survey and the proportion of urban respondents has increased from the first to fifth survey round. Moreover, there has been an improvement in the educational attainment of respondents and their exposure to mass media.

**Table 1:** Sample Characteristics of first cohabitation, first sex and first birth among reproductive aged women (15–49 years) by background characteristics

Background Characteristics	Percentages of First Cohabitation					Percentage of first sex			Percentages of First Birth				
	**NFHS-I**	**NFHS-II**	**NFHS-III**	**NFHS-IV**	**NFHS-V**	**NFHS-III**	**NFHS-IV**	**NFHS-V**	**NFHS-I**	**NFHS-II**	**NFHS-III**	**NFHS-IV**	**NFHS-V**
Current Age													
15–19	10.2	9.2	6.9	3.6	2.8	7.2	4.0	3.2	5.4	4.9	3.4	1.3	1.1
20–24	20.1	18.4	17.4	15.7	13.2	17.8	15.9	13.6	18.6	17.0	15.7	12.5	10.7
25–29	19.5	19.9	19.4	20.1	19.0	19.4	19.9	19.1	20.8	21.0	20.2	19.7	18.8
30–34	16.4	16.9	17.5	17.8	17.7	17.4	17.4	17.5	18.0	18.3	18.7	18.6	18.6
35–39	13.9	14.7	15.9	16.5	17.4	15.6	16.2	17.2	15.3	16.0	17.2	17.8	18.6
40–44	10.9	11.8	13.1	13.7	14.6	12.9	13.6	14.3	12.1	12.9	14.2	15.4	15.7
45–49	9.0	9.2	9.9	12.6	15.3	9.8	13.0	15.0	9.9	10.0	10.7	14.7	16.4
Regions													
East	16.2	17.6	15.3	18.9	17.0	15.2	19.2	17.0	15.9	17.3	15.1	18.7	16.9
West	12.4	11.6	12.9	8.3	10.3	13.0	8.3	10.3	12.5	11.6	12.8	8.3	10.2
North	22.6	23.0	18.5	20.0	19.7	18.6	20.1	19.8	22.8	23.4	18.7	19.9	19.8
South	18.9	17.7	18.9	13.5	16.6	18.6	13.2	16.4	18.9	17.6	18.7	13.6	16.5
Central	19.7	18.0	18.7	26.1	22.6	18.8	26.4	22.7	19.5	17.7	18.7	25.8	22.6
Northeast	10.3	12.2	15.8	13.2	14.0	15.8	12.9	13.8	10.5	12.5	16.1	13.7	14.1
Residence													
Urban	26.2	26.2	30.8	33.4	31.6	31.1	33.7	31.8	26.5	26.5	31.1	33.6	31.6
Rural	73.8	73.8	69.2	66.6	68.4	69.0	66.3	68.2	73.5	73.5	69.0	66.4	68.4
Level of Education													
No Education	61.7	53.4	47.8	33.0	28.2	47.1	32.5	27.4	62.4	54.3	49.1	35.2	29.6
Primary	16.4	16.9	15.5	14.3	14.1	15.5	14.2	13.9	16.3	17.0	15.4	14.8	14.6
Secondary	18.6	21.8	30.9	42.6	45.2	31.5	42.8	45.7	18.1	21.1	30.1	41.1	44.5
Higher	3.4	7.9	5.7	10.2	12.5	6.0	10.4	13.0	3.3	7.6	5.4	8.9	11.4
Caste													
SC	12.1	18.5	19.4	21.1	22.9	19.2	21.1	22.8	12.0	18.5	19.3	21.1	22.8
ST	8.8	8.8	8.5	9.5	9.8	8.5	9.5	9.8	8.8	8.7	8.4	9.5	9.7
OBC		33.2	40.6	45.4	45.2	40.7	45.2	45.2		33.0	40.5	45.3	45.3
Others	79.1	39.5	31.6	24.0	22.1	31.7	24.2	22.3	79.2	39.8	31.8	24.1	22.3
Religion													
Hindu	82.0	81.8	81.5	81.4	82.0	81.5	81.4	82.0	81.8	81.7	81.4	81.4	82.0
Muslim	12.0	12.5	13.2	13.2	13.1	13.1	13.1	13.0	12.0	12.6	13.2	13.1	13.0
Christian	2.4	2.5	2.2	2.2	2.3	2.3	2.3	2.3	2.4	2.6	2.3	2.3	2.2
Others	3.6	3.2	3.07	3.2	2.7	3.1	3.2	2.7	3.7	3.1	3.2	3.2	2.8
Wealth Index													
Poorest	15.9	15.9	12.9	19.8	21.3	12.7	19.8	21.1	15.9	16.8	12.8	20.0	21.4
Poorer	17.2	16.9	15.4	21.6	22.1	15.3	21.4	22.0	17.1	17.6	15.3	21.7	22.2
Middle	20.0	19.7	19.3	20.7	20.9	19.2	20.6	20.8	19.9	20.4	19.4	20.8	20.8
Richer	23.6	22.7	23.5	19.4	19.1	23.4	19.4	19.2	23.6	22.5	23.4	19.4	19.0
Richest	23.3	24.9	29	18.5	16.7	29.5	18.7	17.0	23.5	22.7	29.1	18.2	16.6
Mass Media Exposure													
No	47.3	40.3	26.0	20.6	24.1	25.8	20.4	23.7	47.7	40.8	26.5	20.9	24.5
Any	52.7	59.7	74.0	79.4	75.9	74.2	79.6	76.3	52.3	59.2	73.5	79.1	75.6
Household Structure													
nuclear			50.8	47.9	47.0	50.6	47.5	47.0			53.1	50.2	49.4
non-nuclear			49.2	52.1	53.0	49.5	52.5	53.0			46.9	49.8	50.6
Prior Relationship to Husband													
No				85.7	87.1		85.9	87.1				86.0	87.4
Yes				14.3	12.9		14.1	12.9				14.0	12.7
Total (N)	89,463	90,303	93,724	4,99,615	542,830	89,735	4,87,841	5,25,002	78,969	80,635	84,139	4,72,342	4,91,886

Table 2 depicts the percentage of women who first cohabited by specific exact age and the median age at first cohabitation by current age. This table shows that the percentage of the first cohabitation at exact age 15 in NFHS I is 30.2% and 1.3% in NFHS V, whereas age at first cohabitation has not been considered for the women of age above 15, since while considering the 15–19 as a current age group when we estimate 16 as exact age of cohabitation, we miss all those women who is in age 15 years, similar condition will arise for further ages in same age group. Furthermore, for the same current age group, we were not able to calculate the median age at cohabitation due to the fact that before reaching age 15 less than 50% of respondents completed their first cohabitation. For the age group 20–24 the percentage of the first cohabitation at the exact age 18 in NFHS I is 61.6% and 22.3% in NFHS V, however median age at first cohabitation for the same age group in NFHS I is 17.05 and it increases over the rounds. For the age group 25–29 the percentage of the first cohabitation at exact age 15 in NFHS I is 22.6%, whereas The median age at first cohabitation for the same age group (25–29 years) in NFHS I is 17.00 however it is 19.78 in NFHS V. the percentage of first cohabitation at all exact ages has been declined significantly within last 30 to 35 years. similar decreasing pattern in the percentage of first cohabitation by exact age were found for the age group.

**Table 2:** Distribution of age at first cohabitation by exact age in India over the period 1992–2021

Percentage of women who first cohabited by specific exact age and median age at first cohabitation by current age											
Current Age	NFHS-Rounds	Percentage married by exact age								Number of Women	Median age at first Cohabitation
		15	16	17	18	19	20	21	25		
15–19	NFHS-I	30.2	na	na	na	na	na	na	na	9,098	a
	NFHS-II	29.5	na	na	na	na	na	na	na	8,276	a
	NFHS-III	8.2	na	na	na	na	na	na	na	6,482	a
	NFHS-IV	1.9	na	na	na	na	na	na	na	18,065	a
	NFHS-V	1.3	na	na	na	na	na	na	na	15,324	a
20–24	NFHS-I	21.6	35.8	49.4	61.6	76.2	85.7	na	na	17,979	17.05
	NFHS-II	20.8	33.9	47.1	59.2	74.5	84.1	na	na	16,587	17.24
	NFHS-III	13.1	22.6	33.1	44.5	54.8	63.3	na	na	16,259	18.53
	NFHS-IV	5.4	9.6	16.2	25.3	36.3	46.8	na	na	78,430	a
	NFHS-V	4.0	7.8	13.9	22.3	32.6	42.5	na	na	71,448	a
25–29	NFHS-I	22.6	36.7	50.0	60.4	72.2	79.0	86.0	97.4	17,440	17.00
	NFHS-II	21.7	34.7	48.0	58.4	70.6	78.2	85.1	97.2	17,965	17.19
	NFHS-III	18.6	29.1	41.1	52.1	62.9	71.0	77.5	91.1	18,211	17.81
	NFHS-IV	10.5	16.8	24.8	34.5	44.7	53.9	62.3	83.8	1,00,344	19.52
	NFHS-V	7.7	13.1	20.9	31.1	42.1	52.2	61.0	83.1	1,02,933	19.78
30–34	NFHS-I	22.5	37.3	51.0	61.3	73.3	79.4	86.1	95.9	1,4673	16.93
	NFHS-II	22.0	36.2	50.5	61.4	72.7	79.3	85.6	96.1	15,288	16.97
	NFHS-III	21.3	33.1	46.0	57.3	67.6	74.9	80.8	93.1	16,417	17.35
	NFHS-IV	13.9	21.8	31.4	41.8	51.8	60.4	67.8	85.4	88,810	18.82
	NFHS-V	11.8	19.1	28.0	38.1	48.5	57.7	66.1	87.0	95,988	19.17
35–39	NFHS-I	24.7	39.8	54.2	64.0	75.3	81.2	87.5	95.9	12,466	16.71
	NFHS-II	23.4	37.7	52.1	62.3	73.4	80.2	86.0	95.7	13,252	16.85
	NFHS-III	22.0	34.7	47.8	58.8	69.7	77.5	83.1	93.5	14,861	17.20
	NFHS-IV	15.0	23.2	32.6	43.0	53.1	61.6	68.6	84.9	82,245	18.69
	NFHS-V	13.6	22.4	32.7	43.9	54.7	63.5	71.0	88.9	94,595	18.56
40–44	NFHS-I	27.7	43.1	56.5	66.5	77.7	83.2	89.9	96.8	9,757	16.51
	NFHS-II	24.5	39.3	53.0	63.7	74.9	81.6	88.0	96.7	10,656	16.78
	NFHS-III	22.9	35.4	48.7	59.8	70.3	78.0	83.9	94.5	12,260	17.12
	NFHS-IV	15.8	24.3	33.7	43.6	52.8	60.9	67.9	83.0	68,586	18.69
	NFHS-V	15.2	24.3	34.5	45.8	56.6	65.9	73.7	90.5	79,418	18.39
45–49	NFHS-I	30.0	44.9	58.0	67.7	78.5	84.1	90.4	97.2	8,049	16.39
	NFHS-II	26.0	40.4	54.1	65.3	75.7	81.4	88.1	96.6	8,278	16.70
	NFHS-III	21.2	33.3	46.4	57.8	68.7	77.0	83.2	94.3	9,234	17.32
	NFHS-IV	14.0	22.1	30.3	39.4	48.6	56.7	63.4	78.6	63,135	19.17
	NFHS-V	14.6	23.6	33.9	45.1	56.1	65.3	72.7	90.2	83,124	18.45
15–49	NFHS-I	24.7	na	na	na	na	na	na	na	89,462	a
	NFHS-II	23.3	na	na	na	na	na	na	na	90,303	a
	NFHS-III	17.0	na	na	na	na	na	na	na	93,724	a
	NFHS-IV	10.1	na	na	na	na	na	na	na	4,99,615	a
	NFHS-V	9.0	na	na	na	na	na	na	na	5,42,830	a
20–49	NFHS-I	24.1	38.7	52.3	62.9	75.1	81.9	na	na	80,364	16.83
	NFHS-II	22.7	36.5	50.2	61.1	73.3	80.7	na	na	82,027	16.99
	NFHS-III	19.2	30.5	42.7	53.9	64.5	72.5	na	na	87,242	17.65
	NFHS-IV	11.8	18.8	27.2	37.0	47.0	56.0	na	na	4,81,550	19.33
	NFHS-V	10.6	17.6	26.3	36.6	47.3	56.8	na	na	5,27,506	19.25
25–49	NFHS-I	24.8	39.5	53.1	63.2	74.7	80.9	87.5	96.6	62,385	16.77
	NFHS-II	23.1	37.1	51.0	61.6	73.0	79.9	86.3	96.5	65,440	16.93
	NFHS-III	21.0	32.8	45.6	56.7	67.4	75.2	81.2	93.1	70,983	17.40
	NFHS-IV	13.6	21.3	30.2	40.1	49.9	58.5	65.9	83.4	4,03,120	19.01
	NFHS-V	12.2	20.0	29.4	40.1	50.9	60.3	68.3	87.6	4,56,058	18.92

**Notes**: na = Not applicable, a = Median is not calculated because less than 50% of women had a cohabited before reaching the beginning of the age group

Table 3 demonstrates the distribution of age at first sex by exact age and the median age at first sex in India during NFHS III to NFHS V. From this table, it may be noted that the percentage of having first sex at the age 15 for the age group 15–19 in NFHS III is 7.6%, 1.5% in NFHS IV and 1.1% in NFHS V. Furthermore, for the current age group 20–24, 50.9% decline was found from NFHS III to NFHS V in age at first sex for women at the age of 18. For the age group 20–24 years, median age at first sex is 18.7 during NFHS III. For the age group 25–29, 16.3%% women already had sex at the age of 15 in NFHS III, 6.8% in NFHS IV and 5.6% in NFHS V, for the same age group the percentage of having first sex at the age 25 is 86.8% in NFHS III and 81.1% in NFHS IV and 80.8% in NFHS V however median age of first sex for the same current age group is 18.17 in NFHS III and 19.9 in NFHS V. For other all age groups similar decreasing pattern in the percentage of first sex by exact age from NFHS III to NFHS V were found whereas increasing pattern in the median age at first sex were found.

**Table 3:** Distribution of age at first sex by exact age in India over the period 2005–2021

Percentage of women who first sex by specific exact age and median age at first sex by current age											
Current Age	NFHS-Rounds	Percentage sex by exact age								Number of Women	Median age at first sex
		15	16	17	18	19	20	21	25		
15–19	NFHS-III	7.6	na	na	na	na	na	na	na	6,436	a
	NFHS-IV	1.5	na	na	na	na	na	na	na	19,469	a
	NFHS-V	1.1	na	na	na	na	na	na	na	17,031	a
20–24	NFHS-III	11.7	20.8	31.1	42.2	52.8	61.2	na	na	15,943	18.7
	NFHS-IV	3.4	7.5	13.8	22.7	35.2	46.1	na	na	77,783	a
	NFHS-V	3.0	6.7	12.5	20.7	32.4	42.2	na	na	71,539	a
25–29	NFHS-III	16.3	26.4	38.0	48.2	59.0	66.8	73.3	86.8	17,425	18.17
	NFHS-IV	6.8	12.9	21.0	30.5	42.6	51.9	60.6	81.1	96,924	19.8
	NFHS-V	5.6	10.9	18.5	28.4	41.1	51.0	59.9	80.8	1,00,112	19.9
30–34	NFHS-III	18.1	29.9	42.2	52.9	63.2	70.1	75.9	88.0	15,581	17.73
	NFHS-IV	8.9	17.4	27.2	37.6	49.6	58.2	65.9	82.2	84,845	19.03
	NFHS-V	8.5	15.8	24.8	35.1	47.3	56.4	64.8	83.8	92,083	19.23
35–39	NFHS-III	19.1	31.4	43.9	54.6	65.1	72.3	77.8	87.8	14,025	17.57
	NFHS-IV	9.8	18.7	28.8	39.9	52.3	60.4	68.0	82.4	79,045	18.82
	NFHS-V	10.0	18.8	29.3	40.7	53.4	62.0	69.7	85.7	90,303	18.73
40–44	NFHS-III	19.6	31.8	44.6	55.2	65.7	72.9	78.8	88.8	11,574	17.51
	NFHS-IV	11.0	20.2	30.4	41.1	52.6	60.9	68.6	81.9	66,519	18.77
	NFHS-V	11.2	20.4	30.9	42.3	54.6	63.6	71.6	86.5	75,251	18.63
45–49	NFHS-III	18.3	30.3	43.1	53.7	64.5	72.4	78.6	89.1	8,751	17.65
	NFHS-IV	10.8	20.5	30.3	40.3	51.6	59.8	67.2	81.2	63,255	18.85
	NFHS-V	11.2	20.9	31.7	43.0	55.6	64.2	72.0	87.2	78,683	18.56
15–49	NFHS-III	14.9	na	na	na	na	na	na	na	89,735	a
	NFHS-IV	6.9	na	na	na	na	na	na	na	4,87,841	a
	NFHS-V	6.7	na	na	na	na	na	na	na	5,25,002	a
20–49	NFHS-III	16.6	27.6	39.5	50.2	60.7	68.3	na	na	83,299	17.99
	NFHS-IV	8.0	15.4	24.2	34.2	46.3	55.3	na	na	4,68,371	19.31
	NFHS-V	7.9	14.9	23.7	33.9	46.3	55.5	na	na	5,07,971	19.3
25–49	NFHS-III	18.1	29.7	42.0	52.5	63.1	70.5	76.4	87.9	67,356	17.76
	NFHS-IV	9.2	17.5	27.0	37.3	49.2	57.8	65.6	81.8	3,90,588	19.07
	NFHS-V	9.0	16.9	26.4	37.2	49.7	58.8	67.0	84.5	4,36,432	19.02

**Notes**: na = Not applicable, a = Median is not calculated because less than 50% of women had a done sex before reaching the beginning of the age group

Table 4 demonstrates the age at first birth by exact age in India over the period 1992–2021, from the table it may be noted that the percentage of first birth at exact age 15 for the women of current age group 15–19 is 6.8% in NFHS I and 0.1% in NFHS V. For the age group 20–24, in NFHS I, 34.3% women already had their first birth at the age of 18 which is declined to 8.2% in NFHS V with approx. decrement of 76.1%, whereas the median age at the first birth for the same age group is 19.25 in NFHS 1 and 19.19 in NFHS II. Additionally, Table 4 also depicts that in all the current ages, distribution of age at first birth by exact ages were declined significantly during the period of three decades, whereas during the same period, median ages has increased by approximately 2 years for the age group 25–49 years.

Kaplan Meier failure estimates of age at first cohabitation, first sex, and first birth in India by background factors are shown in Appendix Figures A1, A2, and A3, respectively. This figure shows that the overall age at first cohabitation increased over the years. Among education, women with higher education have greater probability of having higher age at first cohabitation and over the years, the age at cohabitation for women with no education, primary or secondary are shifting towards higher educated women, same pattern can be seen for religion, caste and regions of India that is within all categories of every background characteristic decline in age at first cohabitation can be observed. Similar pattern obtained for age at first sex and age at first birth as well.

**Table 4:** Distribution of age at first birth by exact age in India over the period 1992–2021

Percentage of women who first birth by specific exact age and median age at first birth by current age											
Current Age	NFHS-Rounds	Percentage married by exact age								Number of Women	Median age at first Marriage
		15	16	17	18	19	20	21	25		
15–19	NFHS-I	6.8	na	na	na	na	na	na	na	4,295	a
	NFHS-II	6.8	na	na	na	na	na	na	na	3,926	a
	NFHS-III	1.1	na	na	na	na	na	na	na	2,826	a
	NFHS-IV	0.2	na	na	na	na	na	na	na	6,144	a
	NFHS-V	0.1	na	na	na	na	na	na	na	5,379	a
20–24	NFHS-I	5.9	12.8	22.6	34.3	47.0	59.2	na	na	14,694	19.25
	NFHS-II	6.0	13.5	24.0	35.1	47.6	60.1	na	na	13,697	19.19
	NFHS-III	3.2	7.3	13.1	21.5	30.6	41.4	na	na	13,200	a
	NFHS-IV	0.8	2.1	4.5	9.1	16.1	25.9	na	na	59,046	a
	NFHS-V	0.7	1.8	4.0	8.2	14.7	23.3	na	na	52,787	a
25–29	NFHS-I	5.7	12.7	22.8	34.4	46.5	57.6	67.6	88.6	16,402	19.31
	NFHS-II	5.8	13.8	23.7	35.4	46.6	57.9	67.5	88.7	16,906	19.3
	NFHS-III	4.6	10.3	18.7	28.7	39.4	50.2	59.1	81.6	16,957	19.98
	NFHS-IV	2.0	4.5	8.7	14.9	23.3	33.4	43.6	74.1	92,979	21.68
	NFHS-V	1.4	3.3	6.8	12.7	20.9	31.1	41.7	71.4	92,552	21.86
30–34	NFHS-I	5.3	12.0	21.7	33.2	45.6	57.1	67.4	88.3	14,196	19.38
	NFHS-II	6.0	13.4	23.7	35.6	47.7	58.4	68.3	88.3	14,782	19.21
	NFHS-III	5.0	11.0	19.9	30.1	41.9	53.7	63.0	84.4	15,767	19.69
	NFHS-IV	2.8	5.9	11.3	19.1	28.6	39.5	49.5	77.7	87,727	21.05
	NFHS-V	2.3	4.9	9.6	16.6	25.7	35.9	46.2	76.2	91,532	21.4
35–39	NFHS-I	5.6	12.8	22.7	34.1	46.2	57.1	66.5	87.4	12,053	19.35
	NFHS-II	5.7	12.5	22.8	34.2	46.3	57.1	67.4	87.6	12,903	19.34
	NFHS-III	5.3	11.5	20.8	31.6	42.7	54.0	63.7	85.3	14,428	19.64
	NFHS-IV	3.3	6.8	12.5	20.3	29.9	40.8	51.6	79.3	84,137	20.85
	NFHS-V	2.6	5.8	11.3	19.3	29.3	40.1	50.7	78.4	91,680	20.94
40–44	NFHS-I	5.8	12.6	22.8	34.0	46.5	57.9	68.1	88.8	9,513	19.31
	NFHS-II	6.2	13.7	23.8	35.3	46.9	58.2	67.8	88.0	10,386	19.27
	NFHS-III	4.8	11.4	19.6	30.6	42.7	53.7	63.8	86.0	11,957	19.67
	NFHS-IV	3.6	7.5	13.3	21.6	31.2	41.9	52.5	80.5	72,677	20.77
	NFHS-V	2.9	6.6	12.1	20.1	30.1	40.8	51.4	79.8	77,120	20.87
45–49	NFHS-I	6.5	13.6	23.1	34.2	45.6	56.6	66.0	87.5	7,817	19.4
	NFHS-II	5.3	12.7	21.7	32.4	43.9	55.1	64.7	86.6	8,035	19.54
	NFHS-III	4.1	9.4	16.5	26.5	36.4	47.3	58.5	84.8	9,004	20.25
	NFHS-IV	3.1	6.6	11.9	19.2	28.0	38.3	49.3	78.4	69,632	21.08
	NFHS-V	2.8	6.2	11.5	19.3	28.8	39.7	50.4	79.0	80,835	20.96
15–49	NFHS-I	5.84	na	na	na	na	na	na	na	78,969	a
	NFHS-II	5.96	na	na	na	na	na	na	na	80,635	a
	NFHS-III	3.75	na	na	na	na	na	na	na	84,139	a
	NFHS-IV	2.04	na	na	na	na	na	na	na	4,72,342	a
	NFHS-V	1.68	na	na	na	na	na	na	na	4,91,886	a
20–49	NFHS-I	5.7	12.7	22.6	34.0	46.3	57.7	na	na	74,675	19.32
	NFHS-II	5.9	13.3	23.4	34.9	46.7	58.1	na	na	76,709	19.29
	NFHS-III	4.4	10.0	17.9	27.8	38.5	49.6	na	na	81,313	a
	NFHS-IV	2.4	5.3	9.9	16.7	25.4	35.8	na	na	4,66,198	a
	NFHS-V	2.0	4.5	8.8	15.5	24.2	34.4	na	na	4,86,507	a
25–49	NFHS-I	5.7	12.7	22.6	34.0	46.1	57.3	67.2	88.2	59,981	19.35
	NFHS-II	5.8	13.3	23.3	34.8	46.5	57.5	67.4	88.0	63,012	19.32
	NFHS-III	4.8	10.8	19.3	29.7	40.8	52.0	61.7	84.2	68,113	19.82
	NFHS-IV	2.9	6.1	11.3	18.7	27.9	38.4	48.9	77.7	4,07,152	21.12
	NFHS-V	2.3	5.2	10.0	17.3	26.6	37.1	47.6	76.6	4,33,720	21.25

**Notes**: na = Not applicable, a = Median is not calculated because less than 50% of women had a birth before reaching the beginning of the age group

Figure A4 in the appendix displays a state-specific hierarchical clustered heat map that depicts the likelihood of first birth, first sexual intercourse, and first cohabitation by exact age. Less height in the dendogram indicates that states are more similar to one another, while darker blue and darker red indicate higher and lower probabilities, respectively, of an event not occurring by exact age. Manipur and Nagaland showed the most similar pattern for first cohabitation during the survey year 2019–21, with a Euclidian distance of 0.02, while Karnataka showed the closest pattern as the national estimate for first sex, with a Euclidian distance of 0.025. Additionally, Andhra Pradesh and Telangana demonstrated the most similar pattern for first birth, with a Euclidian distance of 0.014.

Appendix Table B1 depicts the cox proportional hazard model predicting women’s risk of first cohabitation, sex and birth by demographic characteristics. This table shows that all regions have initiated their first birth later than women who belongs to east region similar pattern has been obtained for first cohabitation (Central Region: - AHR: 1.01, 95% CI: 1.01–1.02) as well as for first sex (Central Region: - AHR: 1.01, 95% CI: 1.00-1.02) except for the central region in model 3. Women belonging to rural areas have initiated first cohabitation (AHR: 1.04, 95% CI: 1.04–1.04) earlier, as well as first sex (AHR: 1.04, 95% CI: 1.04–1.05) and first birth (AHR: 1.02, 95% CI: 1.02–1.03) than the urban women. Women having some education or higher education have initiated all three reproductive events later than women with no education. Additionally, over the survey period, a significant decline in age at first cohabitation, first sex and first birth at a particular age of women has been observed in model 3. In comparison to 1992-93 in the period of 2019-21, age at first cohabitation and first birth at a particular age has declined by 23% (AHR: 0.77; CI: 0.76–0.78), and 20% (AHR: 0.80, 95% CI: 0.79–0.80) respectively. Whereas the first sex has been decline by 17% (AHR: 0.83, 95% CI: 0.83–0.84) in the period 2019-21 by the period 2005-06.

The predicted mean at first cohabitation, first sex, and first birth among women aged 15 to 49 is shown in Table 5. This table shows that for all the region, the predicted mean at first cohabitation, first sex and first birth increased throughout the years. Furthermore, in both urban and rural areas increase in predicted mean at reproductive events can be observed, women belong to urban areas have greater value of predicted mean. Women having higher education have higher predicted mean at first cohabitation, sex and birth; similar results can also be seen among Christian, richest, uses any mass media and having non-nuclear family. Additionally, highest increase in predicted mean age at first cohabitation (8.4%), first sex (5.0%) and first birth (7.9%) were found among uneducated women.

**Table 5:** **Multiple classification analysis estimates of Predicted mean at First Cohabitation, First Sex and First Birth among women aged 15–49 years by background characteristics, 1992–2021**

Variables	First Cohabitation					First Sex			First Birth				
State Region	**NFHS-I**	**NFHS-II**	**NFHS-III**	**NFHS-IV**	**NFHS-V**	**NFHS-III**	**NFHS-IV**	**NFHS-V**	**NFHS-I**	**NFHS-II**	**NFHS-III**	**NFHS-IV**	**NFHS-V**
East	16.8 [16.75 16.85]	17.42 [17.37 17.47]	17.55 [17.5 17.61]	18.22 [18.19 18.24]	18.3 [18.28 18.33]	17.65 [17.6 17.71]	18.32 [18.3 18.34]	18.39 [18.37 18.41]	18.79 [18.74 18.85]	19.19 [19.13 19.24]	19.41 [19.35 19.48]	20.37 [20.34 20.39]	20.37 [20.35 20.4]
West	17.67 [17.61 17.73]	17.4 [17.35 17.46]	18.18 [18.12 18.24]	18.44 [18.4 18.48]	18.87 [18.83 18.9]	18.33 [18.27 18.39]	18.53 [18.5 18.56]	18.82 [18.79 18.85]	19.53 [19.47 19.59]	19.22 [19.15 19.28]	20.11 [20.04 20.17]	20.54 [20.51 20.58]	20.83 [20.8 20.87]
North	17.42 [17.37 17.46]	17.66 [17.62 17.7]	17.86 [17.8 17.91]	18.73 [18.7 18.75]	19.27 [19.25 19.29]	18.04 [17.99 18.09]	18.8 [18.77 18.82]	19.3 [19.28 19.33]	19.54 [19.49 19.59]	19.56 [19.52 19.61]	19.97 [19.92 20.03]	20.81 [20.78 20.83]	21.15 [21.12 21.17]
South	17.12 [17.07 17.16]	17.34 [17.3 17.39]	17.82 [17.76 17.87]	18.67 [18.64 18.7]	18.77 [18.74 18.79]	18.05 [18 18.1]	18.96 [18.93 18.99]	18.85 [18.82 18.87]	18.9 [18.85 18.96]	19.07 [19.01 19.12]	19.71 [19.65 19.77]	20.6 [20.57 20.63]	20.62 [20.6 20.65]
Central	16.94 [16.9 16.99]	16.6 [16.55 16.64]	17.48 [17.43 17.53]	18.13 [18.1 18.15]	18.58 [18.56 18.6]	17.56 [17.51 17.61]	18.27 [18.25 18.29]	18.55 [18.54 18.57]	19.21 [19.16 19.27]	18.69 [18.64 18.74]	19.53 [19.47 19.58]	20.42 [20.4 20.44]	20.72 [20.69 20.74]
Northeast	17.6 [17.53 17.67]	18.35 [18.29 18.41]	18.86 [18.79 18.92]	19.46 [19.42 19.5]	19.76 [19.72 19.79]	18.97 [18.9 19.03]	19.59 [19.56 19.62]	19.68 [19.64 19.71]	18.96 [18.88 19.04]	19.69 [19.62 19.76]	20.48 [20.41 20.55]	21.13 [21.09 21.16]	21.26 [21.22 21.29]
Residence													
Urban	17.27 [17.23 17.31]	17.54 [17.5 17.58]	17.93 [17.89 17.97]	18.67 [18.65 18.69]	19.09 [19.07 19.11]	18.08 [18.05 18.12]	18.78 [18.76 18.8]	19.08 [19.06 19.1]	19.17 [19.13 19.22]	19.3 [19.25 19.34]	19.79 [19.75 19.83]	20.69 [20.67 20.71]	20.97 [20.95 21]
Rural	17.19 [17.17 17.22]	17.37 [17.35 17.4]	17.92 [17.89 17.95]	18.47 [18.46 18.48]	18.81 [18.8 18.82]	18.05 [18.02 18.09]	18.61 [18.6 18.62]	18.83 [18.82 18.84]	19.18 [19.15 19.21]	19.2 [19.18 19.23]	19.89 [19.86 19.93]	20.58 [20.57 20.59]	20.76 [20.74 20.77]
Highest Education													
No Education	16.4 [16.37 16.43]	16.51 [16.48 16.54]	16.75 [16.71 16.79]	17.56 [17.54 17.58]	17.77 [17.75 17.79]	16.93 [16.89 16.97]	17.72 [17.7 17.74]	17.78 [17.76 17.8]	18.58 [18.54 18.61]	18.53 [18.5 18.57]	18.93 [18.89 18.97]	20.01 [19.99 20.03]	20.04 [20.02 20.06]
Primary	17.11 [17.06 17.16]	17.06 [17.01 17.1]	17.11 [17.05 17.17]	17.73 [17.7 17.76]	17.93 [17.91 17.96]	17.27 [17.21 17.32]	17.91 [17.89 17.94]	17.98 [17.96 18.01]	18.99 [18.93 19.04]	18.85 [18.8 18.9]	19.13 [19.07 19.19]	19.92 [19.9 19.95]	20.02 [20 20.05]
Secondary	18.55 [18.51 18.6]	18.22 [18.18 18.27]	18.55 [18.51 18.59]	18.9 [18.88 18.91]	19.13 [19.12 19.15]	18.61 [18.57 18.65]	18.98 [18.97 19]	19.12 [19.1 19.13]	20.17 [20.11 20.22]	19.79 [19.74 19.84]	20.33 [20.28 20.37]	20.82 [20.8 20.83]	20.94 [20.92 20.95]
Higher	21.68 [21.58 21.78]	20.95 [20.88 21.02]	22.15 [22.07 22.24]	21.85 [21.81 21.88]	22.09 [22.06 22.13]	22.14 [22.06 22.22]	21.83 [21.79 21.86]	21.98 [21.95 22.01]	23.18 [23.06 23.3]	22.49 [22.41 22.58]	23.74 [23.65 23.83]	23.67 [23.63 23.71]	23.8 [23.76 23.83]
Caste													
SC	16.97 [16.91 17.03]	17.17 [17.13 17.22]	17.64 [17.59 17.7]	18.39 [18.37 18.42]	18.71 [18.69 18.73]	17.81 [17.75 17.86]	18.54 [18.52 18.56]	18.73 [18.71 18.75]	18.85 [18.79 18.92]	18.92 [18.86 18.97]	19.51 [19.46 19.57]	20.43 [20.41 20.45]	20.62 [20.59 20.64]
ST	17.23 [17.17 17.3]	17.28 [17.22 17.34]	17.78 [17.71 17.86]	18.67 [18.64 18.69]	19.18 [19.15 19.2]	17.87 [17.8 17.94]	18.79 [18.76 18.81]	19.15 [19.12 19.17]	18.89 [18.81 18.96]	18.87 [18.8 18.95]	19.51 [19.43 19.59]	20.58 [20.55 20.61]	20.93 [20.91 20.96]
OBC	17.25 [17.23 17.27]	17.44 [17.4 17.47]	17.81 [17.77 17.85]	18.45 [18.43 18.47]	18.76 [18.75 18.78]	17.96 [17.92 18]	18.56 [18.55 18.58]	18.77 [18.76 18.79]	19.27 [19.25 19.3]	19.31 [19.27 19.35]	19.82 [19.78 19.86]	20.6 [20.59 20.62]	20.76 [20.75 20.78]
Others		17.56 [17.53 17.59]	18.21 [18.17 18.25]	18.68 [18.66 18.7]	19 [18.98 19.02]	18.36 [18.32 18.4]	18.83 [18.81 18.85]	19.04 [19.01 19.06]		19.41 [19.37 19.45]	20.16 [20.11 20.2]	20.81 [20.79 20.83]	20.97 [20.95 21]
Religion													
Hindu	17.06 [17.04 17.08]	17.34 [17.32 17.36]	17.86 [17.83 17.88]	18.41 [18.39 18.42]	18.71 [18.7 18.72]	18.01 [17.98 18.03]	18.56 [18.55 18.57]	18.74 [18.73 18.75]	19.09 [19.06 19.11]	19.2 [19.18 19.23]	19.84 [19.81 19.86]	20.55 [20.54 20.56]	20.7 [20.69 20.71]
Muslim	16.72 [16.66 16.78]	16.98 [16.92 17.03]	17.47 [17.41 17.54]	18.55 [18.52 18.58]	19.33 [19.3 19.37]	17.61 [17.54 17.68]	18.69 [18.66 18.72]	19.34 [19.3 19.37]	18.48 [18.41 18.55]	18.57 [18.51 18.64]	19.18 [19.11 19.26]	20.4 [20.37 20.44]	20.99 [20.96 21.03]
Christian	19.29 [19.2 19.37]	18.96 [18.87 19.04]	18.96 [18.86 19.06]	19.35 [19.3 19.4]	19.75 [19.7 19.8]	19.01 [18.92 19.11]	19.18 [19.13 19.22]	19.61 [19.57 19.66]	21 [20.9 21.1]	20.59 [20.48 20.69]	20.69 [20.58 20.79]	21.19 [21.14 21.24]	21.38 [21.33 21.43]
Others	17.94 [17.85 18.04]	18.21 [18.12 18.3]	18.43 [18.33 18.54]	19.31 [19.26 19.36]	19.52 [19.47 19.57]	18.59 [18.48 18.69]	19.44 [19.4 19.49]	19.44 [19.4 19.48]	19.59 [19.49 19.7]	19.8 [19.69 19.91]	20.2 [20.09 20.31]	21.19 [21.14 21.24]	21.32 [21.27 21.37]
Wealth Index													
Poorest	16.74 [16.68 16.8]	16.97 [16.91 17.02]	17.49 [17.42 17.57]	18.44 [18.41 18.47]	18.84 [18.82 18.87]	17.63 [17.56 17.7]	18.54 [18.52 18.57]	18.83 [18.8 18.85]	18.83 [18.77 18.9]	18.86 [18.79 18.92]	19.45 [19.37 19.53]	20.68 [20.65 20.71]	20.83 [20.8 20.86]
Poor	16.89 [16.84 16.94]	17.01 [16.96 17.06]	17.49 [17.43 17.55]	18.27 [18.25 18.3]	18.69 [18.66 18.71]	17.6 [17.54 17.66]	18.4 [18.38 18.42]	18.7 [18.68 18.72]	18.87 [18.81 18.93]	18.87 [18.81 18.93]	19.4 [19.34 19.47]	20.4 [20.38 20.42]	20.65 [20.62 20.67]
Middle	17.04 [16.99 17.08]	17.22 [17.17 17.26]	17.71 [17.66 17.76]	18.38 [18.35 18.4]	18.76 [18.74 18.79]	17.84 [17.79 17.89]	18.51 [18.49 18.53]	18.78 [18.76 18.8]	18.99 [18.94 19.04]	19.04 [18.99 19.09]	19.64 [19.59 19.7]	20.43 [20.41 20.46]	20.69 [20.67 20.71]
Richer	17.37 [17.33 17.41]	17.63 [17.59 17.67]	18.06 [18.01 18.1]	18.64 [18.62 18.67]	18.95 [18.92 18.97]	18.2 [18.15 18.24]	18.79 [18.76 18.81]	18.97 [18.95 18.99]	19.26 [19.22 19.31]	19.35 [19.31 19.4]	19.92 [19.87 19.97]	20.65 [20.63 20.68]	20.84 [20.82 20.87]
Richest	17.72 [17.67 17.78]	17.98 [17.93 18.03]	18.38 [18.33 18.43]	18.96 [18.93 18.99]	19.23 [19.2 19.26]	18.54 [18.49 18.59]	19.09 [19.06 19.11]	19.25 [19.22 19.28]	19.64 [19.58 19.7]	19.75 [19.69 19.81]	20.33 [20.27 20.38]	20.94 [20.91 20.97]	21.1 [21.07 21.13]
Mass Media Exposure													
No	17.22 [17.18 17.25]	17.43 [17.39 17.47]	17.84 [17.78 17.89]	18.53 [18.5 18.55]	18.83 [18.81 18.85]	18 [17.95 18.06]	18.64 [18.61 18.66]	18.81 [18.79 18.83]	19.26 [19.22 19.3]	19.33 [19.28 19.37]	19.9 [19.84 19.96]	20.76 [20.74 20.79]	20.83 [20.81 20.85]
Any	17.21 [17.19 17.24]	17.42 [17.4 17.45]	17.94 [17.92 17.97]	18.53 [18.51 18.54]	18.89 [18.88 18.91]	18.08 [18.06 18.11]	18.66 [18.65 18.67]	18.91 [18.9 18.92]	19.11 [19.08 19.14]	19.18 [19.15 19.21]	19.83 [19.81 19.86]	20.57 [20.55 20.58]	20.8 [20.79 20.81]
Family Structure													
Nuclear			17.88 [17.85 17.91]	18.49 [18.48 18.51]	18.84 [18.83 18.86]	18.01 [17.98 18.04]	18.61 [18.6 18.62]	18.83 [18.82 18.84]			19.89 [19.86 19.92]	20.69 [20.68 20.71]	20.89 [20.87 20.9]
Non-Nuclear			17.97 [17.94 18.01]	18.56 [18.54 18.57]	18.91 [18.9 18.93]	18.13 [18.1 18.16]	18.7 [18.69 18.71]	18.94 [18.93 18.95]			19.79 [19.76 19.83]	20.53 [20.51 20.54]	20.73 [20.71 20.74]
Prior Relationship with husband													
No				18.57 [18.56 18.58]	18.92 [18.91 18.93]		18.69 [18.68 18.7]	18.93 [18.92 18.94]				20.65 [20.64 20.66]	20.85 [20.84 20.86]
Yes				18.22 [18.18 18.25]	18.54 [18.51 18.57]		18.38 [18.35 18.41]	18.55 [18.52 18.58]				20.32 [20.29 20.36]	20.5 [20.47 20.53]
Total	**17.21 [17.19 17.24]**	**17.42 [17.4 17.44]**	**17.96 [17.93 17.98]**	**18.55 [18.54 18.56]**	**18.89 [18.88 18.9]**	**18.1 [18.07 18.12]**	**18.68 [18.67 18.69]**	**18.91 [18.9 18.92]**	**19.17 [19.15 19.2]**	**19.23 [19.2 19.25]**	**19.87 [19.84 19.89]**	**20.63 [20.62 20.64]**	**20.81 [20.8 20.82]**

**Table 6:** Multivariate Decomposition result showing the percentage contribution of background characteristics which leads to overall change in age at first Cohabitation, First Sex* and First Birth among reproductive aged women in India, 1992–2021

Background Characteristics	First Cohabitation								First Sex								First Birth							
	Due to difference in Characteristics (E)				Due to difference in Coefficients (C)				Due to difference in Characteristics (E)				Due to difference in Coefficients (C)				Due to difference in Characteristics (E)				Due to difference in Coefficients (C)			
	Coef.	SE	P-value	Percent Contribution	Coef.	SE	P-value	Percent Contribution	Coef.	SE	P-value	Percent Contribution	Coef.	SE	P-value	Percent Contribution	Coef.	SE	P-value	Percent Contribution	Coef.	SE	P-value	Percent Contribution
**Age**				**-9.4**				**52.5**				**11.3**				**51.2**				**-15.9**				**58.1**
15–19																								
20–24	-0.037	0.001	0.000	-2.4	0.066	0.010	0.000	4.3	-0.026	0.001	0.000	-3.6	0.054	0.011	0.000	7.7	-0.053	0.002	0.000	-4.0	0.076	0.015	0.000	5.8
25–29	-0.073	0.001	0.000	-4.7	0.151	0.010	0.000	9.8	-0.006	0.000	0.000	-0.9	0.082	0.010	0.000	11.7	-0.104	0.002	0.000	-7.9	0.140	0.016	0.000	10.6
30–34	-0.071	0.001	0.000	-4.6	0.162	0.009	0.000	10.5	-0.010	0.000	0.000	-1.4	0.075	0.009	0.000	10.6	-0.107	0.002	0.000	-8.1	0.164	0.013	0.000	12.4
35–39	-0.020	0.000	0.000	-1.3	0.159	0.008	0.000	10.3	0.013	0.000	0.000	1.9	0.061	0.008	0.000	8.7	-0.033	0.000	0.000	-2.5	0.153	0.011	0.000	11.6
40–44	-0.003	0.000	0.000	-0.2	0.130	0.006	0.000	8.4	0.016	0.000	0.000	2.2	0.049	0.007	0.000	6.9	-0.005	0.000	0.000	-0.4	0.117	0.009	0.000	8.8
45–49	0.058	0.001	0.000	3.8	0.144	0.006	0.000	9.3	0.093	0.001	0.000	13.2	0.040	0.005	0.000	5.6	0.092	0.001	0.000	7.0	0.118	0.008	0.000	8.9
**Education**				**62.9**				**-12.1**				**40.6**				**-35.8**				**65.4**				**-10.1**
No Education																								
Primary	-0.018	0.001	0.000	-1.2	-0.073	0.006	0.000	-4.7	-0.008	0.000	0.000	-1.2	-0.026	0.005	0.000	-3.6	-0.013	0.001	0.000	-1.0	-0.047	0.006	0.000	-3.6
Secondary	0.520	0.004	0.000	33.6	-0.096	0.007	0.000	-6.2	0.128	0.001	0.000	18.1	-0.128	0.015	0.000	-18.1	0.439	0.004	0.000	33.2	-0.075	0.008	0.000	-5.7
Higher	0.471	0.002	0.000	30.5	-0.019	0.003	0.000	-1.2	0.168	0.001	0.000	23.7	-0.100	0.005	0.000	-14.1	0.438	0.002	0.000	33.2	-0.011	0.003	0.000	-0.8
**Mass Media**				**0.5**				**1.4**				**-0.7**				**-8.5**				**-1.0**				**3.6**
No																								
Any	0.008	0.003	0.006	0.5	0.021	0.044	0.631	1.4	-0.005	0.001	0.000	-0.7	-0.060	0.064	0.345	-8.5	-0.013	0.003	0.000	-1.0	0.047	0.049	0.331	3.6
**Caste**				**1.4**				**-7.2**				**2.2**				**-14.8**				**0.0**				**-9.8**
SC																								
ST	0.033	0.001	0.000	2.2	0.022	0.006	0.000	1.4	0.023	0.001	0.000	3.3	0.040	0.007	0.000	5.7	0.022	0.001	0.000	1.6	0.028	0.006	0.000	2.1
Others	-0.012	0.002	0.000	-0.8	-0.134	0.026	0.000	-8.7	-0.008	0.001	0.000	-1.1	-0.145	0.023	0.000	-20.5	-0.022	0.002	0.000	-1.6	-0.157	0.029	0.000	-11.9
**Religion**				**0.6**				**1.4**				**-2.4**				**13.8**				**0.4**				**1.9**
Hindu																								
Muslim	-0.001	0.000	0.000	0.1	0.105	0.004	0.000	6.8	-0.007	0.000	0.000	-1.1	0.102	0.005	0.000	14.5	-0.001	0.000	0.000	0.0	0.094	0.004	0.000	7.1
Christian	0.008	0.000	0.000	0.5	-0.078	0.004	0.000	-5.0	-0.010	0.000	0.000	-1.4	-0.008	0.005	0.101	-1.1	0.005	0.000	0.000	0.4	-0.072	0.004	0.000	-5.5
Others	0.001	0.000	0.000	0.0	-0.005	0.003	0.049	-0.3	0.001	0.000	0.000	0.1	0.003	0.003	0.275	0.4	0.000	0.000	0.000	0.0	0.003	0.003	0.240	0.3
**Wealth Index**				**-1.5**				**-31.8**				**-7.2**				**-43.8**				**0.7**				**-35.8**
Poorest																								
Poorer	-0.012	0.001	0.000	-0.8	-0.062	0.006	0.000	-4.0	-0.013	0.001	0.000	-1.9	-0.018	0.006	0.005	-2.5	-0.017	0.001	0.000	-1.3	-0.062	0.007	0.000	-4.7
Middle	-0.003	0.000	0.000	-0.2	-0.095	0.008	0.000	-6.1	-0.003	0.000	0.000	-0.4	-0.055	0.009	0.000	-7.8	-0.006	0.000	0.000	-0.5	-0.095	0.008	0.000	-7.2
Richer	0.003	0.001	0.000	0.2	-0.151	0.010	0.000	-9.8	0.000	0.001	0.726	0.0	-0.101	0.012	0.000	-14.3	0.013	0.001	0.000	1.0	-0.144	0.011	0.000	-10.9
Richest	-0.011	0.002	0.000	-0.7	-0.183	0.013	0.000	-11.8	-0.035	0.003	0.000	-4.9	-0.135	0.017	0.000	-19.1	0.019	0.002	0.000	1.4	-0.173	0.015	0.000	-13.1
**Residence**				**-0.9**				**-17.9**				**-5.8**				**-45.7**				**-0.8**				**-24.7**
Urban																								
Rural	-0.013	0.001	0.000	-0.9	-0.277	0.051	0.000	-17.9	-0.041	0.003	0.000	-5.8	-0.323	0.045	0.000	-45.7	-0.010	0.001	0.000	-0.8	-0.327	0.057	0.000	-24.7
**State Regions**				**0.3**				**6.8**				**-5.2**				**15.9**				**0.6**				**-1.0**
East																								
West	-0.012	0.001	0.000	-0.8	-0.043	0.005	0.000	-2.8	-0.011	0.001	0.000	-1.5	-0.029	0.006	0.000	-4.1	-0.008	0.001	0.000	-0.6	-0.043	0.006	0.000	-3.3
North	-0.021	0.000	0.000	-1.4	0.082	0.009	0.000	5.3	0.021	0.000	0.000	2.9	0.095	0.008	0.000	13.4	-0.017	0.000	0.000	-1.3	0.005	0.010	0.632	0.4
South	-0.008	0.001	0.000	-0.5	-0.005	0.007	0.535	-0.3	-0.007	0.000	0.000	-1.0	0.009	0.008	0.243	1.3	-0.001	0.001	0.021	0.1	-0.006	0.008	0.459	-0.5
Central	0.010	0.001	0.000	0.7	0.014	0.007	0.057	0.9	0.006	0.001	0.000	0.8	0.051	0.007	0.000	7.2	0.013	0.001	0.000	1.0	-0.030	0.008	0.000	-2.3
Northeast	0.035	0.001	0.000	2.3	0.057	0.005	0.000	3.7	-0.046	0.001	0.000	-6.5	-0.013	0.008	0.126	-1.8	0.019	0.001	0.000	1.4	0.061	0.006	0.000	4.6
**Constant**					0.823	0.094	0.000	53.2					0.955	0.102	0.000	134.9					0.907	0.116	0.000	68.7
**Total**				**53.9**				**46.1**				**32.7**				**67.3**				**49.2**				**50.8**

**Note**: * indicates the change in first sex was done for the period 2005 to 2021

Appendix figure A5 shows specific predicted mean age at first cohabitation, first sex and first birth among women aged 15–49 years by survey rounds. This figure shows that in NFHS I, there was only one state Goa which has mean age at first cohabitation greater than 21 whereas in NFHS V, 6 states belong to this category. Similarly, for age at first sex, the number of states having predicted mean value greater than 21 is 1 in NFHS I and this number increased to 5 in NFHS V with lowest in Andaman and Nicobar (17.63) and highest in Ladakh (22.41). For age at first cohabitation the number of states having predicted mean age lies between 18 and 21 are 29 in NFHS I which declined to 15 in NFHS V.

The decomposition analysis model takes into consideration the differences in characteristics (compositional factors) as well as differences caused by the effect of characteristics (Table 6). The overall multivariate decomposition analysis result shows that nearly 54% and 50% of the overall increase in mean age at cohabitation and first birth from the period 1992 to 2021 was due to the difference in characteristics respectively. Furthermore, it was found to be 32.7% for the first sex from the period 2005 to 2021. Among the compositional factors, the majority portion of the increase in age at cohabitation during both surveys was explained by education (62.9%) followed by caste (1.4%) and religion (0.6%) respectively. While for the first sex, compositional factors show that education (40.6%), age (11.3%), and caste (2.2%) accounted for the majority of the increase in age at first sex for the period between 2015 and 2021. In a similar manner, for first births, education (65.4%), wealth (0.7%), and regions of India (0.6%) collectively account for the majority of the increase in first birth age from 1992 to 2021. After controlling the effect of compositional factors, approximately 46%, 67% and 50% of change in mean age at first cohabitation, first sex and first birth was due to the difference in the effect of characteristics respectively.

## Discussion

The purpose of this paper is to examine the age pattern of Indian women at first cohabitation, first sex and first birth. Our analysis from various rounds of NFHS data of Indian women showed a measurable change in their reproductive life events over the past three decades. Among education, women with higher education have greater probability of having higher age at first cohabitation. Over the years, the age at cohabitation for women with no education, primary or secondary are shifting towards higher educated women, same pattern can be seen for religion, caste and regions of India. Additionally, within all categories of every background characteristic increase in age at first cohabitation can be observed. Similar pattern was obtained for age at first sex and age at first birth as well. Taking only age at first cohabitation into an account, we found that among women of 15–49, the percentage of women who were already cohabited by exact age declined over the survey round, whereas with the increase in age group, this percentage increased for the first cohabitation over all the survey round. Since women of higher age groups belong to more orthodox and compact social environments, in addition, most of the women of the sample belong to women of higher age groups [34].

Earlier, in India, the marriage customs were not leading to the instant beginning of cohabitation, at that time, childhood marriages were more common and there was a tradition of marriages of girls before she reached puberty. Childhood marriages were socially functional. They have to transfer the woman from her father’s home to the home of her husband and this process is referred as two-stage marriage. Despite all this, the age of cohabitation was too earlier in older times. But with time, this custom of two-stage marriages was dropped and women’s education, labor force participation, freedom of movement and decision became the intermediate variables which results in an increase of age at cohabitation throughout the survey [3, 9].

In all rounds of the survey, women of east and central region started cohabitation earlier. Considering the educational background, women with secondary and higher education delayed their age of first cohabitation, but the propensity to initiate cohabitation of women having no education or primary education seems to increase over time. Among religion, Muslims had cohabitation sooner in NFHS I, II and III after that they had cohabitation later than women of the Hindu religion; changes in behaviors and attitudes related to family and living arrangements and the exposure to different possible lifestyles have given rise to these variations [35]. Also, women of other religions started cohabitation later than women of Hindu and Muslim religions. It was also found that women of the poorest and poor categories started cohabitation sooner than the middle, richer or richest women in all five rounds of the survey. Women who had any exposure to mass media cohabited sooner than women who had none in NFHS I and NFHS II after that woman having any exposure to mass media cohabited later; subsequently, there have been differences in age at cohabitation among race, region, religion educational background, states and union territories [36]. Therefore, one of the principal conclusions drawn from this study is that the term cohabitation has a different concept and meaning in India than it does in western countries. However, it’s changing gradually over time, since India has been slowly and gradually moving towards the western culture by replicating their ideas and lifestyles. Cohabitation has been emerging as a common pattern with distinct reasons among people in Western World. These may include testing their compatibility or establishing financial security before marrying [37]. Despite the fact that cohabitation has been a practice for a very long time, current patterns are fundamentally distinct from earlier ones. Today’s youth has been practicing this concept which has not only become popular among the social and legal researchers but also the historians, to reach whether such a concept or similar setting was prevalent at any time in human civilization. A researcher has further elaborated the cohabitation or non-marital relationships as societal transformation or change in the history of human relationship developments [38].

Our study has identified various correlates of age at first sex or initiation of sexual activities. Findings implied that those who were out of school or never had schooling or those residing in rural areas, having no mass media exposure, nuclear family size and having any kind of prior relationship with husband were more likely to have initiated sex early than others. In several studies, it was found that an increase in educational attainment and an increase in labor force participation among women leads to an increase in the age at initiation of first birth and also causes an increase in the windows between first sex and first birth [39]. Our study findings suggest that women belonging to scheduled tribes and scheduled castes were more likely to initiate reproductive events earlier than other castes. It may be attributed to a tendency among families of young women belonging to other castes to confront to social norms that leads to giving high value to family honor as a symbol and as a result, women faced severe restriction on their freedom of movement and ability to interact with men which forth caused late initiation of sexual activity [40]. Initiation of sexual intercourse may also vary by marriage age. Women marrying at young age in India have little choice in spouse selection and generally have no prior intimate relationship with their spouse [41]. In this study, finding suggests that women in northeast region-initiated sex later than women of other regions, this may be because of the existence matriarchy society in northeast region of India, which results in higher age at first sex. Reproductive health events and their patterns have long been characterized by ethnic and racial differentials and our findings show shreds of evidence of them. Since the effective implementation of family planning policies in the south, the diffusion process, status and autonomy are the reason behind the delay in the initiation of sex [42].

In this study, it was found that the predicted mean at first birth increased over the years, highest value can be observed for northeast region, which is 21.26 in NFHS V. In NFHS I and NFHS III, the higher predicted mean at first birth obtained in women belonging to rural areas. Whereas for the remaining rounds of the survey, higher value has been obtained in urban areas. Women who do not have any mass media exposure have higher predicted mean at first birth for all rounds of NFHS. Women in India have age at first birth relatively lower than other developed countries. Over the past three decades, there has been a noticeable rise in the mean age for first cohabitation, first sex, and first birth. Educated women prefer delay of entry into marriage and they delay childbearing as well, especially in southern states where it is normalized to delay childbearing after marriage [43] Thus, education could play a major role in delaying entry into marriage and this delay could lead to delayed entry into motherhood. Educated women may have greater matters of importance regarding fertility timing than less educated women and therefore, they are expected to delay their first birth [44]. Also, educated women are more likely to engage in paid employment which may compete with motherhood and lead to the postponement of first birth [45, 46]. Additionally, from the multivariate decomposition analysis it was also found that education played the largest contribution among the compositional factors in the overall increase in mean age at key reproductive events such as age at first cohabitation, first sex and first birth. Considering the role of education and the timing of first birth, it appears that much of the influence of education on the timing of first birth is through a delay into marriage rather than a delay in entry into motherhood after marriage. Delays in childbearing may also be motivated by various other factors, not merely education but including higher opportunity costs [47] and a shift from the traditional norm to individual-oriented values and ideologies [48].

State-specific predicted mean age at first cohabitation, first sex and first birth among women aged 15–49 years by survey round found that in NFHS I, there was only one state Goa which has mean age at first cohabitation greater than 21 because of predomination of Christian population and urbanization [9]. whereas in NFHS V, six states belong to this category. Similarly, the number of states belong to women who had the occurrence of these events at age 18 or above increased over the rounds.

## Conclusion

Though reproductive health has for long held an important event in women’s life but they are still very confined to a certain domain. Consequentially India does have some specific policies which dictate the legal age for the occurrence of these events but does not dictate safeguards to regulate those behavior and other related factors that affect those events. Over time the government has formulated several useful legislative measures relating to various domains of reproductive events but given the large size and heterogeneity in social and cultural norms results in changing ideas and choices regarding the initiation of reproductive events henceforth, it has been realized that formulation of nationwide policy needed to be improved or amended. The concern of policy maker is all about the prevalence of earlier sex, cohabitation or first birth and its impact on women and their children’s health status. A substantial proportion of women in India still have cohabitation, first sex and first birth at young age. Enforcing the legal minimum age for all these reproductive events is one action that the government adopted to increase the safeguard of women’s health. In spite of that, special efforts are needed in areas where other factors lead to force women or to make choices about to have cohabitation, sex or first birth at young age. In addition, improving women’s status, age of cohabitation, first sex and first birth at young age will probably bring new socio-economic and health risks and in results these risks may create challenges for service providers and policymakers who are trying to meet young women’s reproductive health care services.

## Electronic supplementary material

Below is the link to the electronic supplementary material.

**Additional File**: Transition in the Ages at Key Reproductive Events and its determinants in India: Evidence from NFHS 1992–93 to 2019–21

## Data Availability

NFHS data is a nationally representative data set which available freely in public domain. For more details, visit at www.measuredhs.com and can be accessed after the request is made to and approved by the DHS program.

## References

[1] Worku F, Gebresilassie S. (2008). Reproductive health for health science students. *Ethiopian Public Health Training Institute, University of Gondar*.

[2] Finer LB, Philbin JM (2014). Trends in ages at key reproductive transitions in the United States, 1951–2010. Women’s Health Issues.

[3] Bongaarts J, Mensch BS, Blanc AK (2017). Trends in the age at reproductive transitions in the developing world: the role of education. Popul Stud.

[4] dos Santos Silva I, Beral V (1997). Socio-economic differences in reproductive behaviour. IARC Sci Publ.

[5] InterLACE Study Team (2019). Variations in reproductive events across life: a pooled analysis of data from 505 147 women across 10 countries. Hum Reprod.

[6] Marphatia AA, Saville NM, Amable GS, Manandhar DS, Cortina-Borja M, Wells JC, Reid AM (2020). How much education is needed to delay women’s age at marriage and first pregnancy?. Front public health.

[7] Ibarra-Nava I, Choudhry V, Agardh A (2020). Desire to delay the first childbirth among young, married women in India: a cross-sectional study based on national survey data. BMC Public Health.

[8] Acharya AK (2010). The influence of female age at marriage on fertility and child loss in India. Trayectorias.

[9] Bloom DE, Reddy PH. (1986). Age patterns of women at marriage, cohabitation, and first birth in India.Demography,509–523. 3803653

[10] Singh S, Samara R. (1996). Early marriage among women in developing countries.International family planning perspectives,148–175.

[11] Ju DH, Okigbo KA, Yim SS, Hardie JH (2022). Ethnic and generational differences in partnership patterns among Asians in the United States. J Ethnic Migration Stud.

[12] Manning WD, Brown SL, Payne KK (2014). Two decades of stability and change in age at first union formation. J Marriage Family.

[13] Manning WD (2020). Young adulthood relationships in an era of uncertainty: a case for cohabitation. Demography.

[14] Bumpass L, Lu HH (2000). Trends in cohabitation and implications for children s family contexts in the United States. Popul Stud.

[15] Bumpass LL, Sweet JA, Cherlin A. (1991). The role of cohabitation in declining rates of marriage.Journal of Marriage and the Family,913–927.

[16] Kaestle CE, Halpern CT, Miller WC, Ford CA (2005). Young age at first sexual intercourse and sexually transmitted infections in adolescents and young adults. Am J Epidemiol.

[17] Louie KS, De Sanjose S, Diaz M, Castellsague X, Herrero R, Meijer CJ, Bosch FX (2009). Early age at first sexual intercourse and early pregnancy are risk factors for cervical cancer in developing countries. Br J Cancer.

[18] Kotchick BA, Shaffer A, Miller KS, Forehand R (2001). Adolescent sexual risk behavior: a multi-system perspective. Clin Psychol Rev.

[19] Robson K, Berthoud R (2003). Teenage motherhood in Europe: a multi-country analysis of socio-economic outcomes. Eur Sociol Rev.

[20] Srivastava S, Sakkarnaikar FS, THE RIGHT OF MAINTENANCE OF WOMEN LIVING IN UNMARRIED COHABITATION IN INDIA. Lex Humana (ISSN 2175 – 0947). 2023;15(1):324–38. AN ANALYSIS OF.

[21] Lichter DT, Qian Z (2008). Serial cohabitation and the marital life course. J Marriage Family.

[22] Raley RK. (2000). Recent trends and differentials in marriage and cohabitation: The United States. *The ties that bind: Perspectives on marriage and cohabitation*, 19–39.

[23] Field E, Ambrus A (2008). Early marriage, age of menarche, and female schooling attainment in Bangladesh. J Polit Econ.

[24] Carmichael S (2011). Marriage and power: age at first marriage and spousal age gap in lesser developed countries. The History of the Family.

[25] Chari AV, Heath R, Maertens A, Fatima F (2017). The causal effect of maternal age at marriage on child wellbeing: evidence from India. J Dev Econ.

[26] Forrest JD, Singh S. (1990). The sexual and reproductive behavior of American women, 1982–1988. *Family planning perspectives*, 206–214. 2272379

[27] McLanahan S, Percheski C (2008). Family structure and the reproduction of inequalities. Annu Rev Sociol.

[28] Musick K (2007). Cohabitation, non-marital childbearing, and the marriage process. Demographic Res.

[29] International Institute for Population Sciences (1995). India: National Family Health Survey (MCH and Family Planning), India 1992-93.

[30] International Institute for Population Sciences (IIPS) and ORC Macro (2000). National Family Health Survey (NFHS-2), 1998–99: India.

[31] International Institute for Population Sciences (IIPS) and Macro International (2007). National Family Health Survey(NFHS-3), 2005–06: India: volume I.

[32] International Institute for Population Sciences (IIPS) and ICF (2017). National Family Health Survey (NFHS-4), 2015-16: India.

[33] International Institute for Population Sciences (IIPS) and ICF (2021). National Family Health Survey (NFHS-5), 2019-21: India.

[34] McPherson JM, Smith-Lovin L (1982). Women and weak ties: differences by sex in the size of voluntary organizations. Am J Sociol.

[35] Dommaraju P (2012). Marriage and fertility dynamics in India. Asia-Pacific Popul J.

[36] Goswami B (2012). An investigation into the pattern of delayed marriage in India.

[37] Sassler S, Lichter DT (2020). Cohabitation and marriage: complexity and diversity in union-formation patterns. J Marriage Family.

[38] Blossfeld HP. (1995). Changes in the process of family formation and women’s growing economic independence: A comparison of nine countries.The new role of women: Family formation in modern societies,3–32.

[39] Goldin C, Katz LF (2002). The power of the pill: oral contraceptives and women’s career and marriage decisions. J Polit Econ.

[40] Srinivas MN (1956). A note on sanskritization and westernization. J Asian Stud.

[41] Mensch BS, Singh S, Casterline JB. (2005). Trends in the timing of first marriage among men and women in the developing world. *The changing transitions to adulthood in developing countries: Selected studies*, 118–171.

[42] Dommaraju P, Agadjanian V (2009). India’s north–south divide and theories of fertility change. J Popul Res.

[43] Jones GW. (2017). Changing marriage patterns in Asia. Routledge handbook of Asian demography (pp. 351–369).Routledge.

[44] Nath DC, Land KC, Goswami G (1999). Effects of the status of women on the first-birth interval in indian urban society. J Biosoc Sci.

[45] Sullivan R (2005). The age pattern of first-birth rates among US women: the bimodal 1990s. Demography.

[46] Edwards ME. (2002, September). Education and occupations: reexamining the conventional wisdom about later first births among American mothers. In Sociological Forum (Vol. 17, 3,pp. 423–443).Kluwer Academic Publishers-Plenum Publishers.

[47] Yu WH (2005). Changes in women’s postmarital employment in Japan and Taiwan. Demography.

[48] Caldwell JC (2005). On net intergenerational wealth flows: an update. Popul Dev Rev.

