# Supplementary material for: Transition in the ages at key reproductive events and its determinants in India: evidence from NFHS 1992-93 to 2019-21
Source: BMC Womens Health. 2023 Mar 29;23:145. doi: 10.1186/s12905-023-02271-w (PMC10061699; doi:10.1186/s12905-023-02271-w)
Supplement: Supplementary file 1 — Additional File: Transition in the Ages at Key Reproductive Events and its determinants in India: Evidence from NFHS 1992–93 to 2019–21 [file 12905_2023_2271_MOESM1_ESM.docx]

**Transition in the Ages at Key Reproductive Events and its determinants in India: Evidence from NFHS 1992-93 to 2019-21**

**Appendix**

**Figure A1:** Kaplan Meier Failure estimates of age at First Cohabitation in India by background characteristics

| **A1.** Overall (2005-06)  **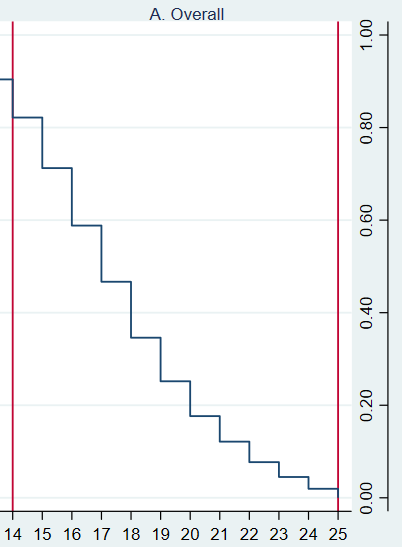** | **A2.** Overall (2015-16)  **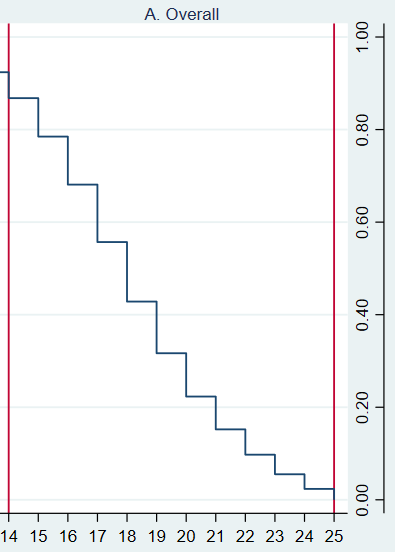** | **A3.** Overall (2019-21)  **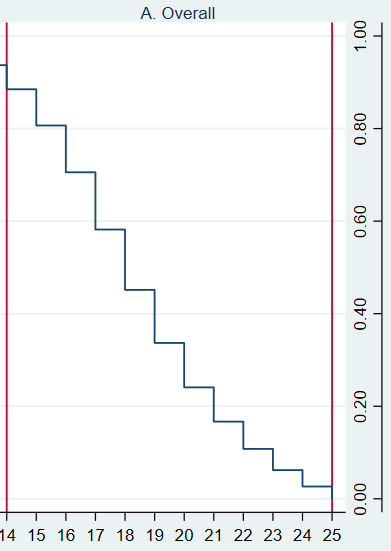** |
| --- | --- | --- |
| **B1.** Education (2005-06)  **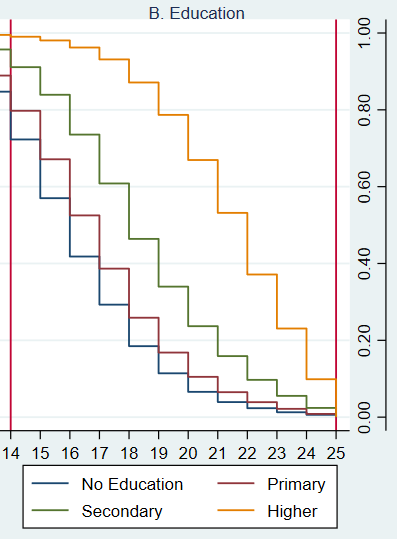** | **B2.** Education (2015-16)  **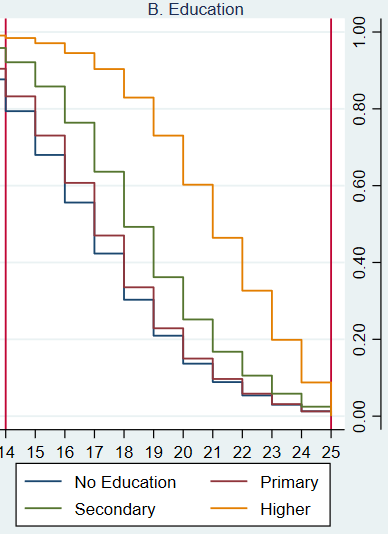** | **B3.** Education (2019-21)  **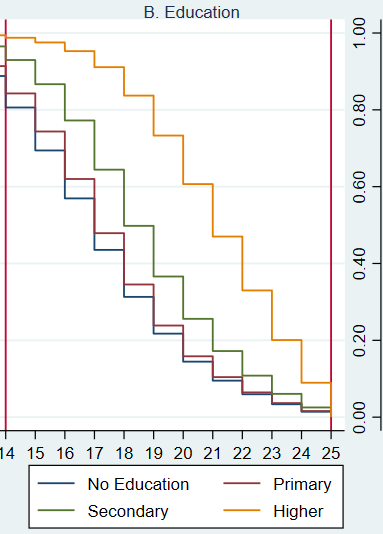** |
| **C1.** Religion (2005-06)  **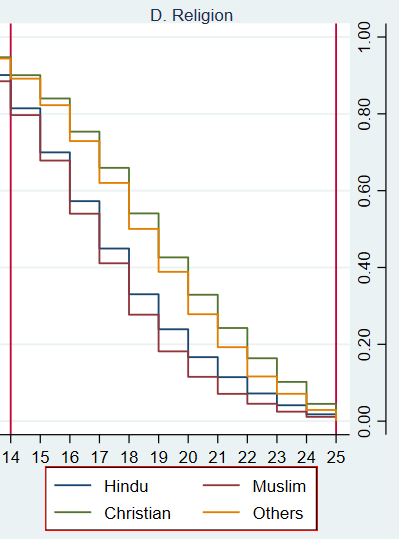** | **C2.** Religion (2015-16)  **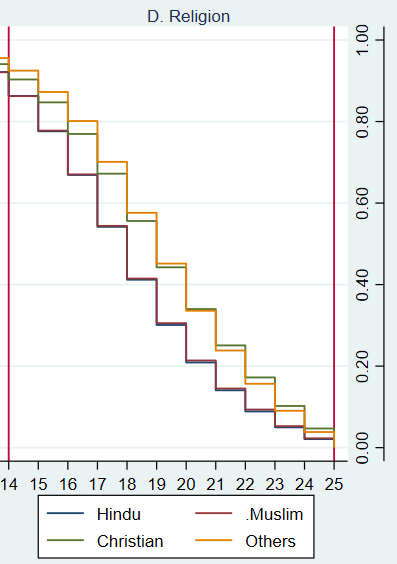** | **C3.** Religion (2019-21)  **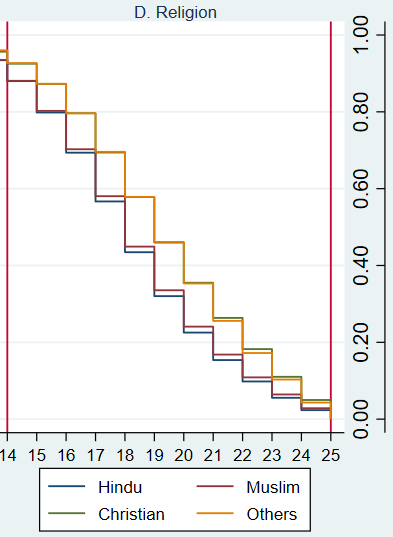** |
| **D1.** Caste (2005-06)  **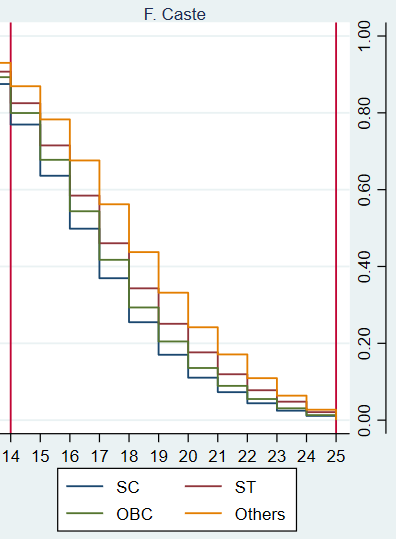** | **D2.** Caste (2015-16)  **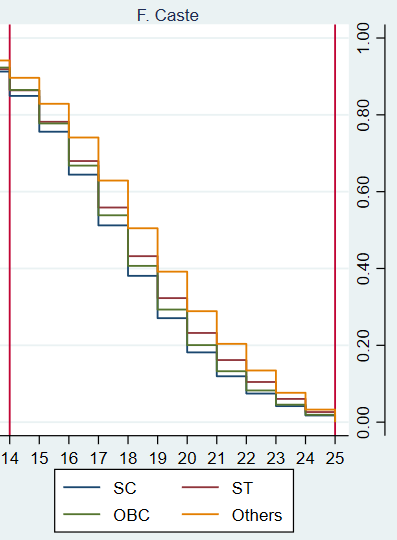** | **D3.** Caste (2019-21)  **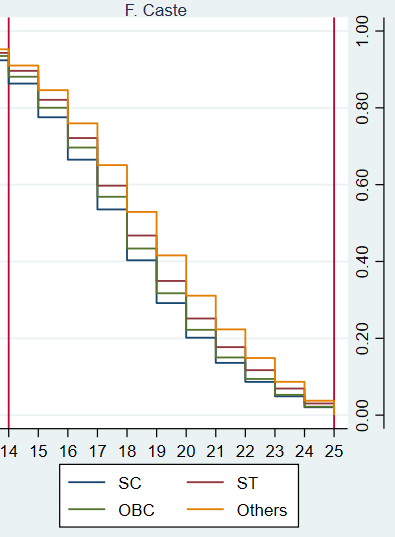** |
| **E1.** Regions of India (2005-06)  **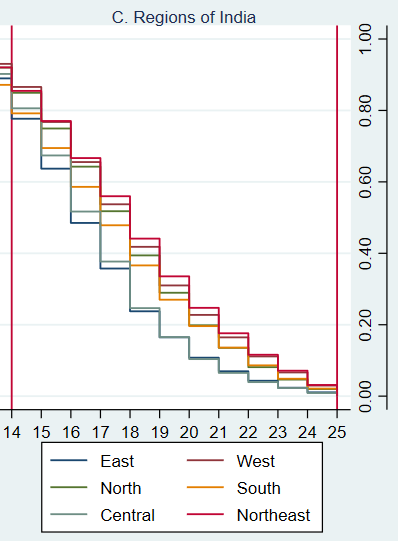** | **E2.** Regions of India (2015-16)  **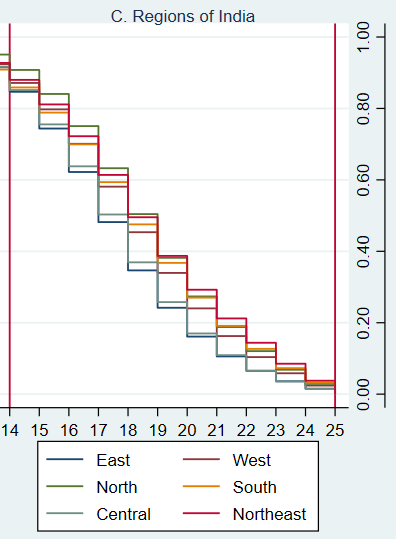** | **E3.** Regions of India (2019-21)  **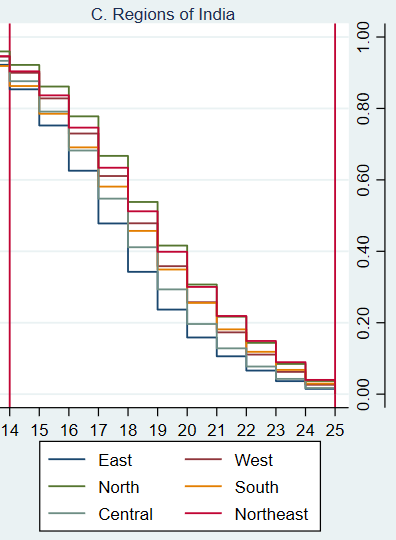** |
| **F1.** Residence (2005-06)  **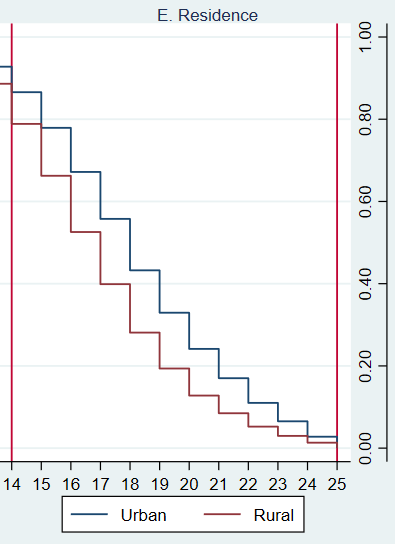** | **F2.** Residence (2015-16)  **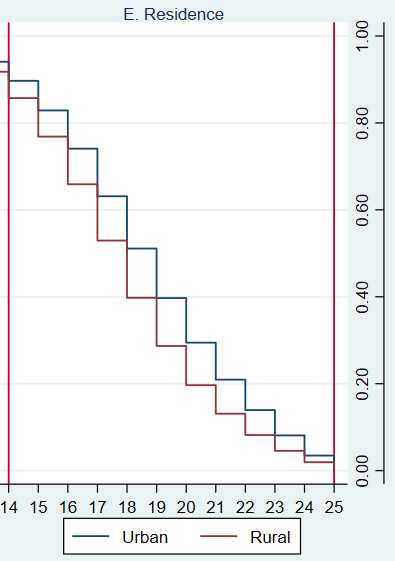** | **F3.** Residence (2019-21)  **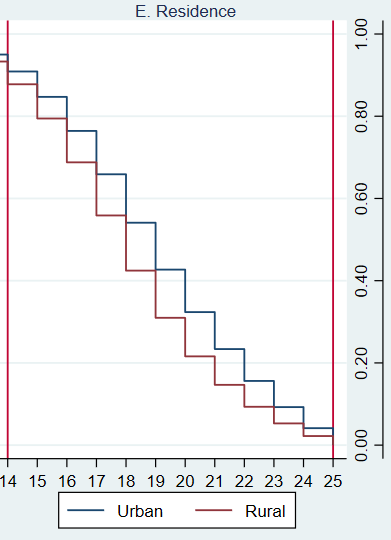** |

**Figure A2:** Kaplan Meier Failure estimates of age at First Sex in India by background characteristics

| A1. Overall (2005-06)  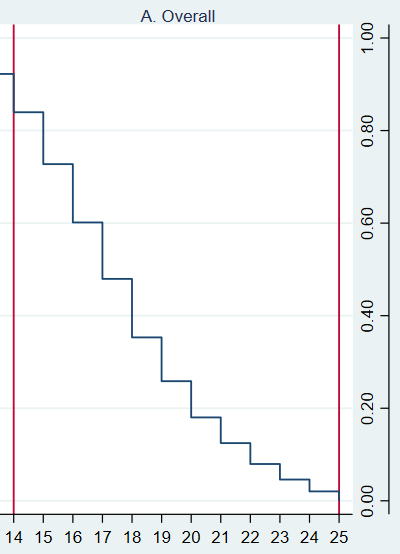 | A2. Overall (2015-16)  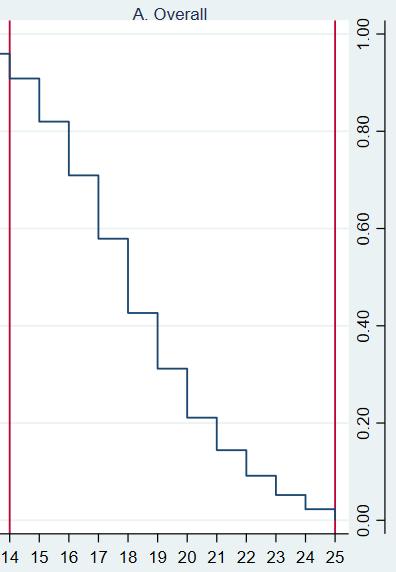 | A3. Overall (2019-21)  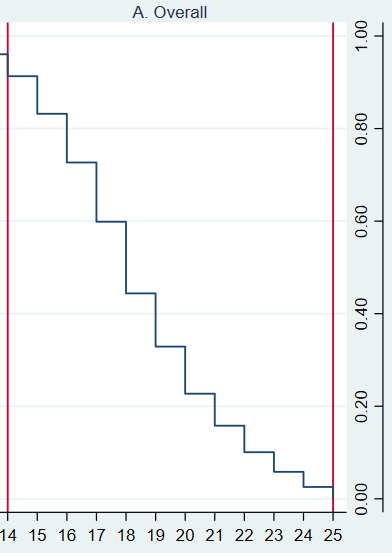 |
| --- | --- | --- |
| B1. Education (2005-06)  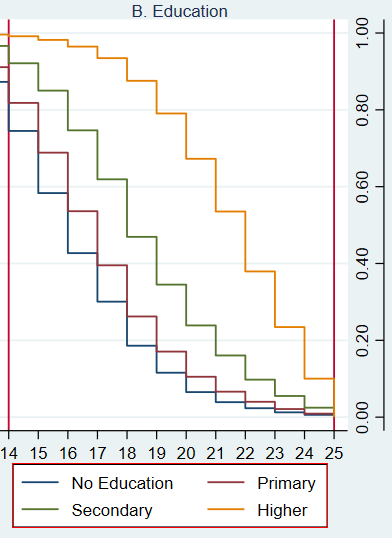 | B2. Education (2015-16)  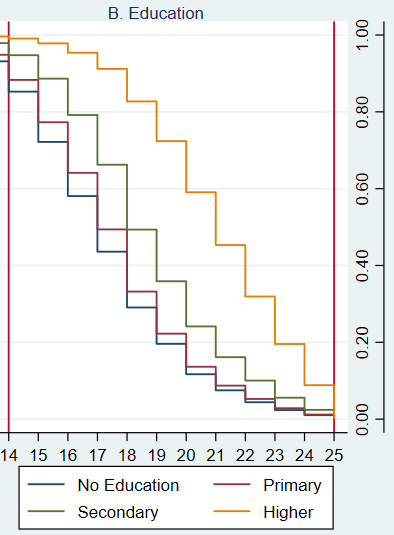 | B3. Education (2019-21)  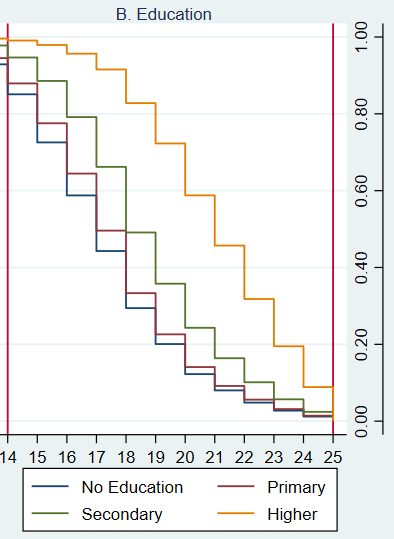 |
| C1. Religion (2005-06)  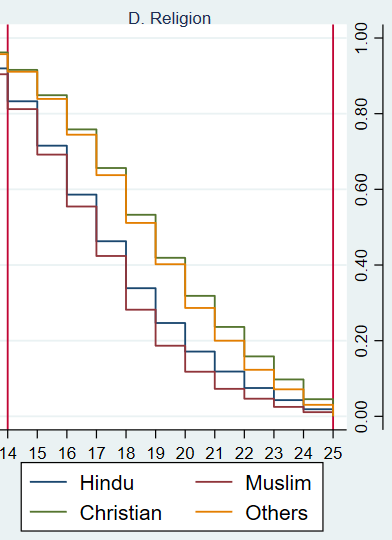 | C2. Religion (2015-16)  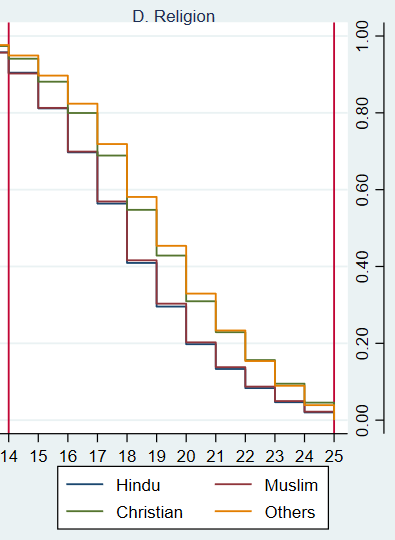 | C3. Religion (2019-21)  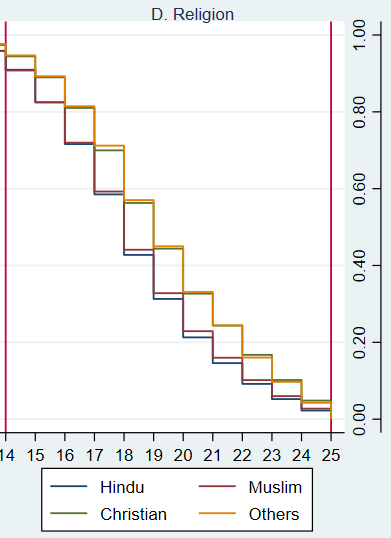 |
| D1. Caste (2005-06)  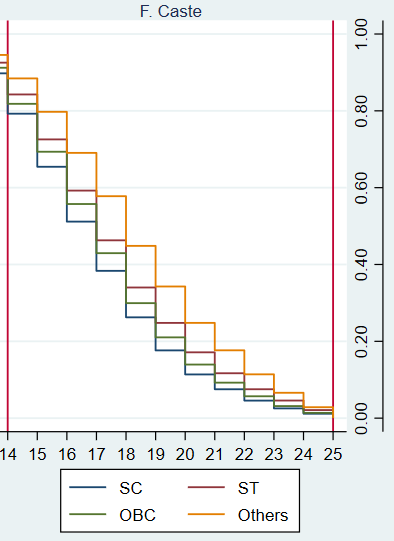 | D2. Caste (2015-16)  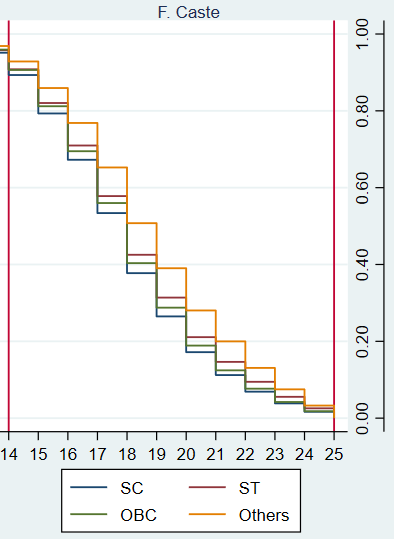 | D3. Caste (2019-21)  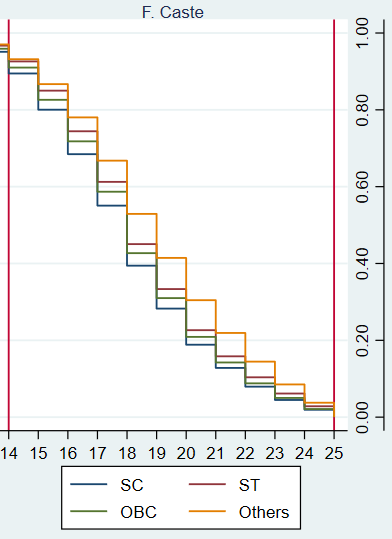 |
| E1. Regions of India (2005-06)  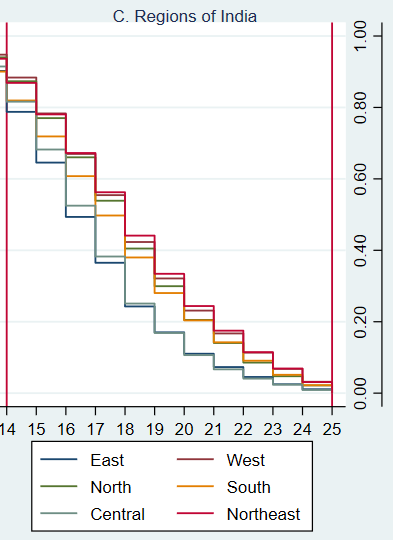 | E2. Regions of India (2015-16)  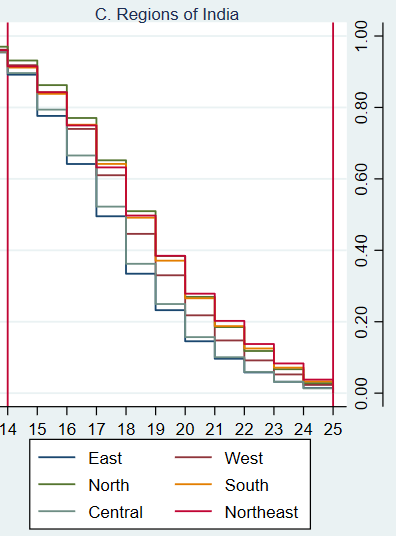 | E3. Regions of India (2019-21)  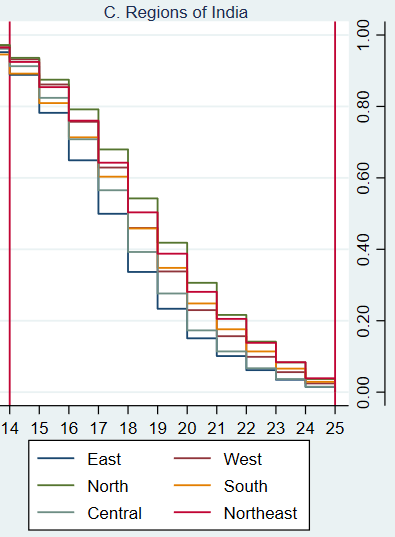 |
| F1. Residence (2005-06)  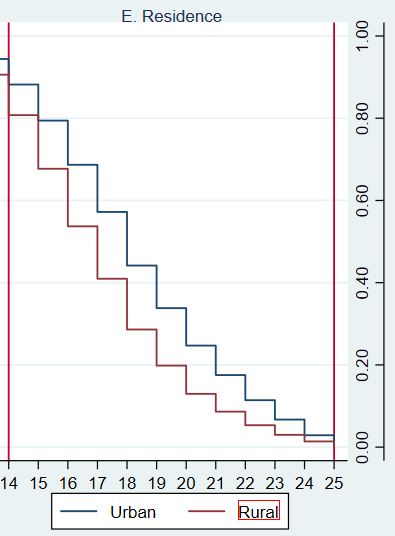 | F2. Residence (2015-16)  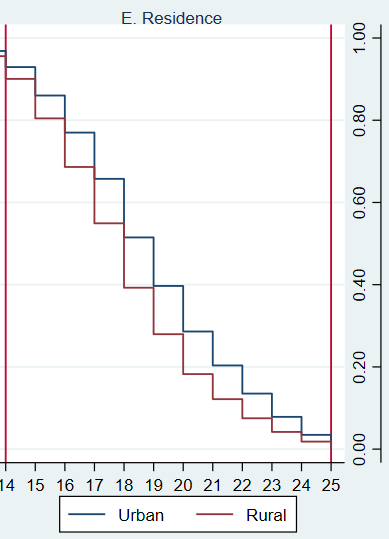 | F3. Residence (2019-21)  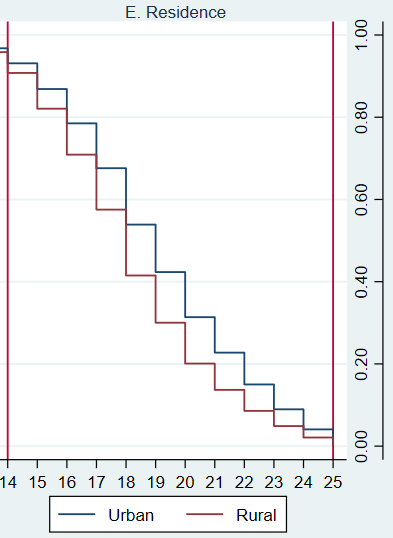 |

**Figure A3:** Kaplan Meier Failure estimates of age at First Birth in India by background characteristics

| A1. Overall (2005-06)  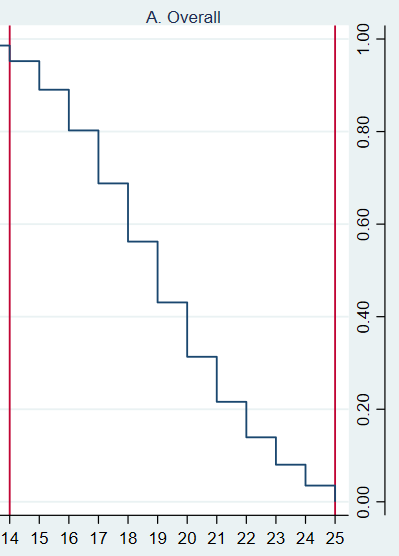 | A2. Overall (2015-16)  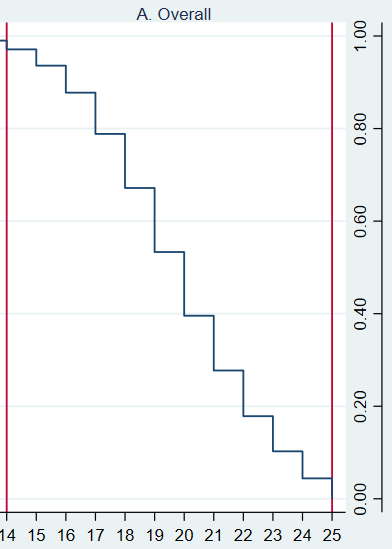 | A3. Overall (2019-21)  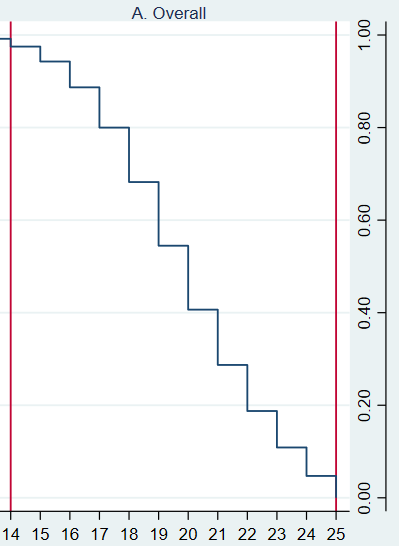 |
| --- | --- | --- |
| B1. Education (2005-06)  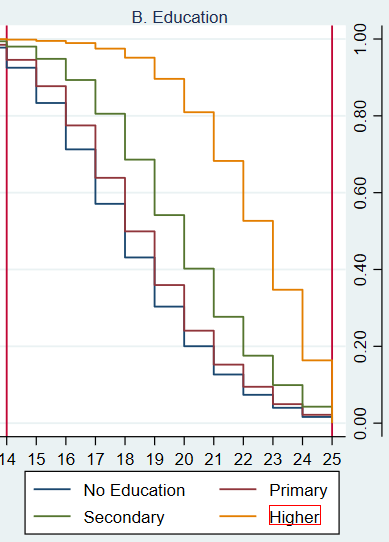 | B2. Education (2015-16)  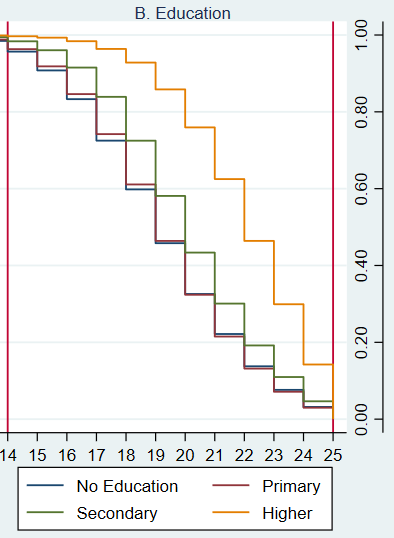 | B3. Education (2019-21)  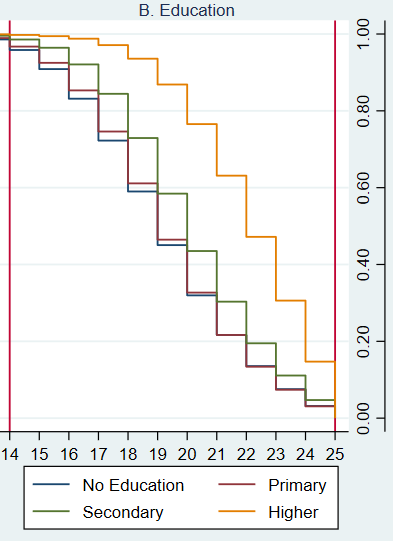 |
| C1. Religion (2005-06)  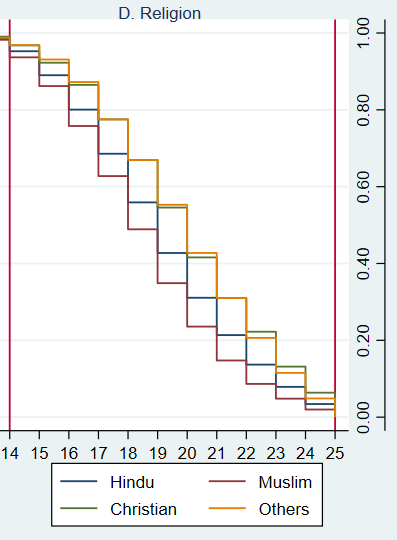 | C2. Religion (2015-16)  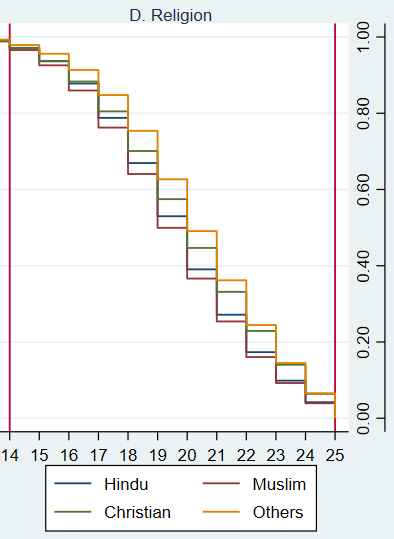 | C3. Religion (2019-21)  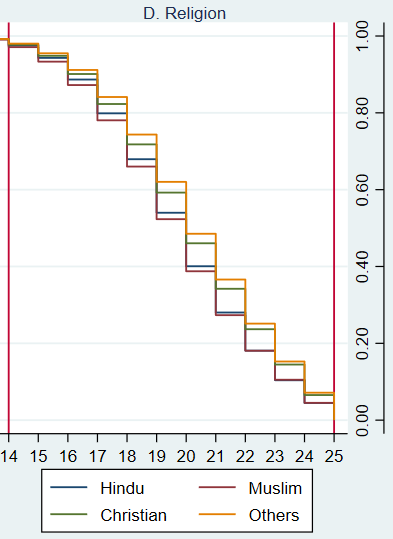 |
| D1. Caste (2005-06)  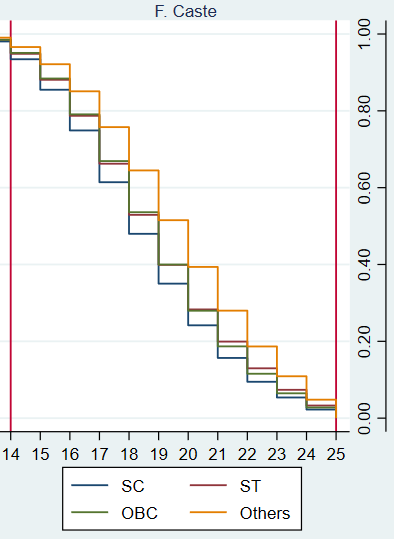 | D2. Caste (2015-16)  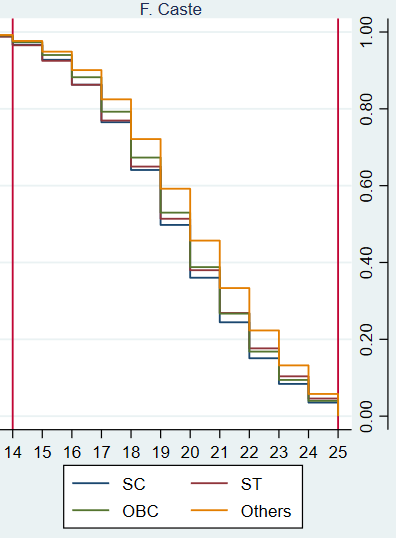 | D3. Caste (2019-21)  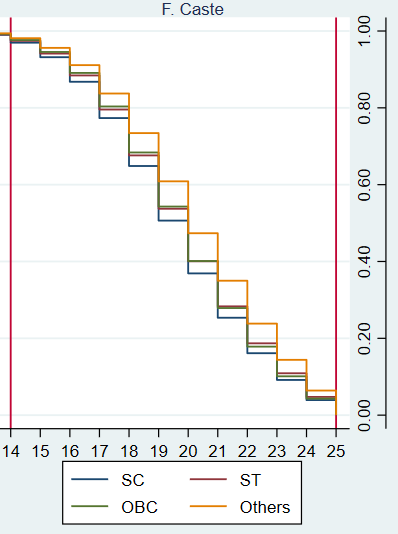 |
| E1. Regions of India (2005-06)  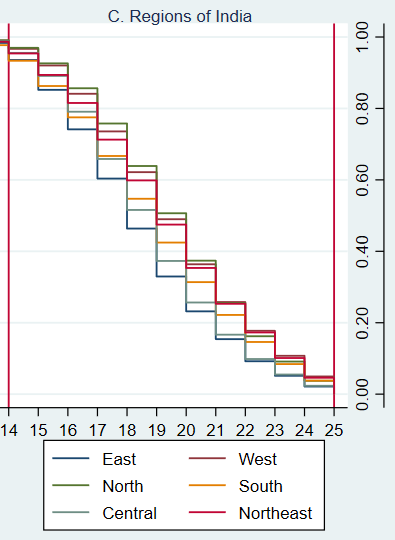 | E2. Regions of India (2015-16)  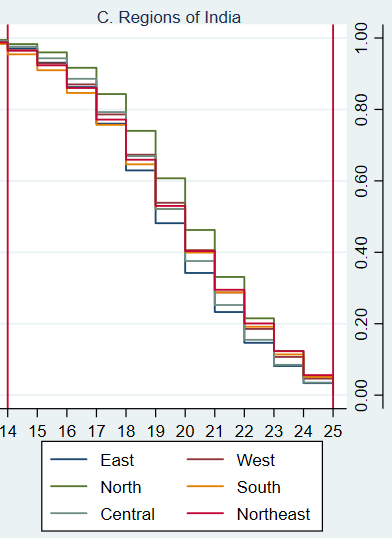 | E3. Regions of India (2019-21)  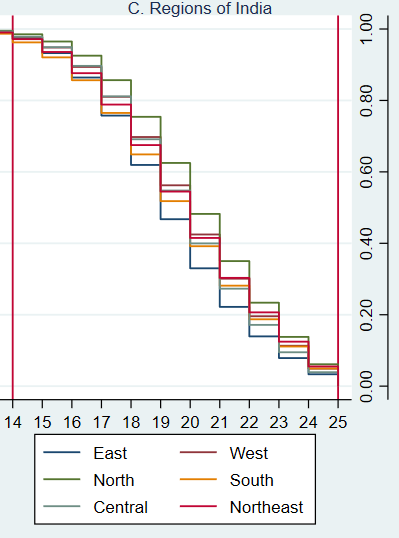 |
| F1. Residence (2005-06)  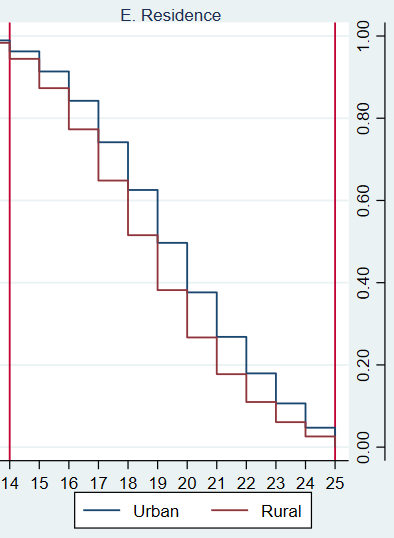 | F2. Residence (2015-16)  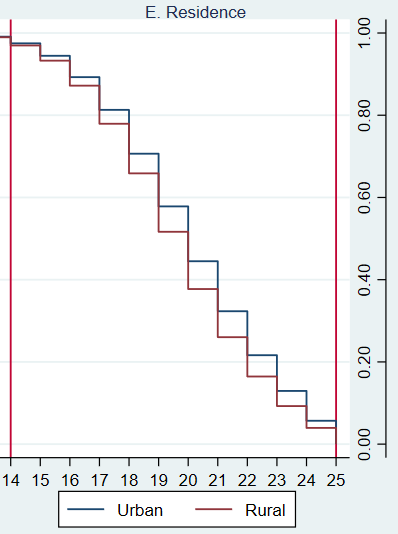 | F3. Residence (2019-21)  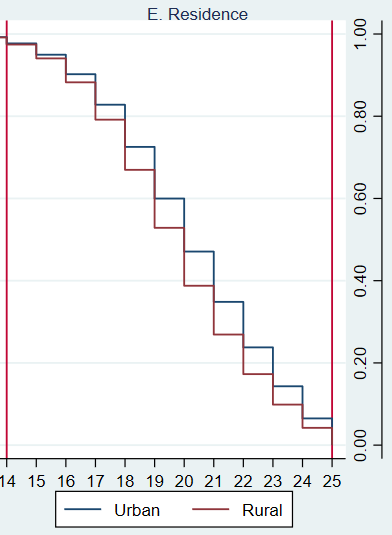 |

**Figure A4:** State specific hierarchical clustered heat map showing the probability of First Cohabitation, First Sex and First Birth not yet happened by exact age among women aged 15-49 years for the last 3 survey rounds.

| I. 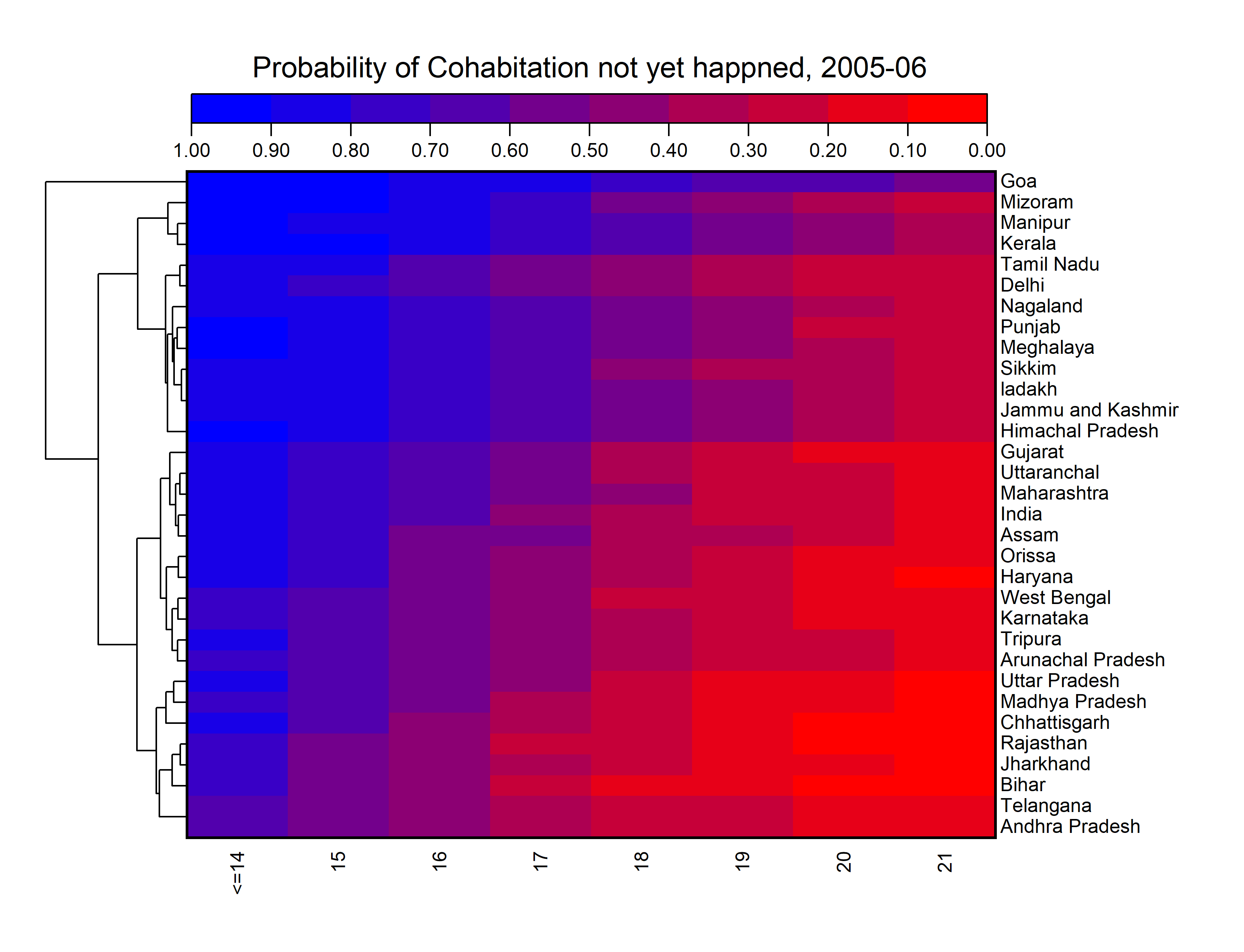 |
| --- |
| II. 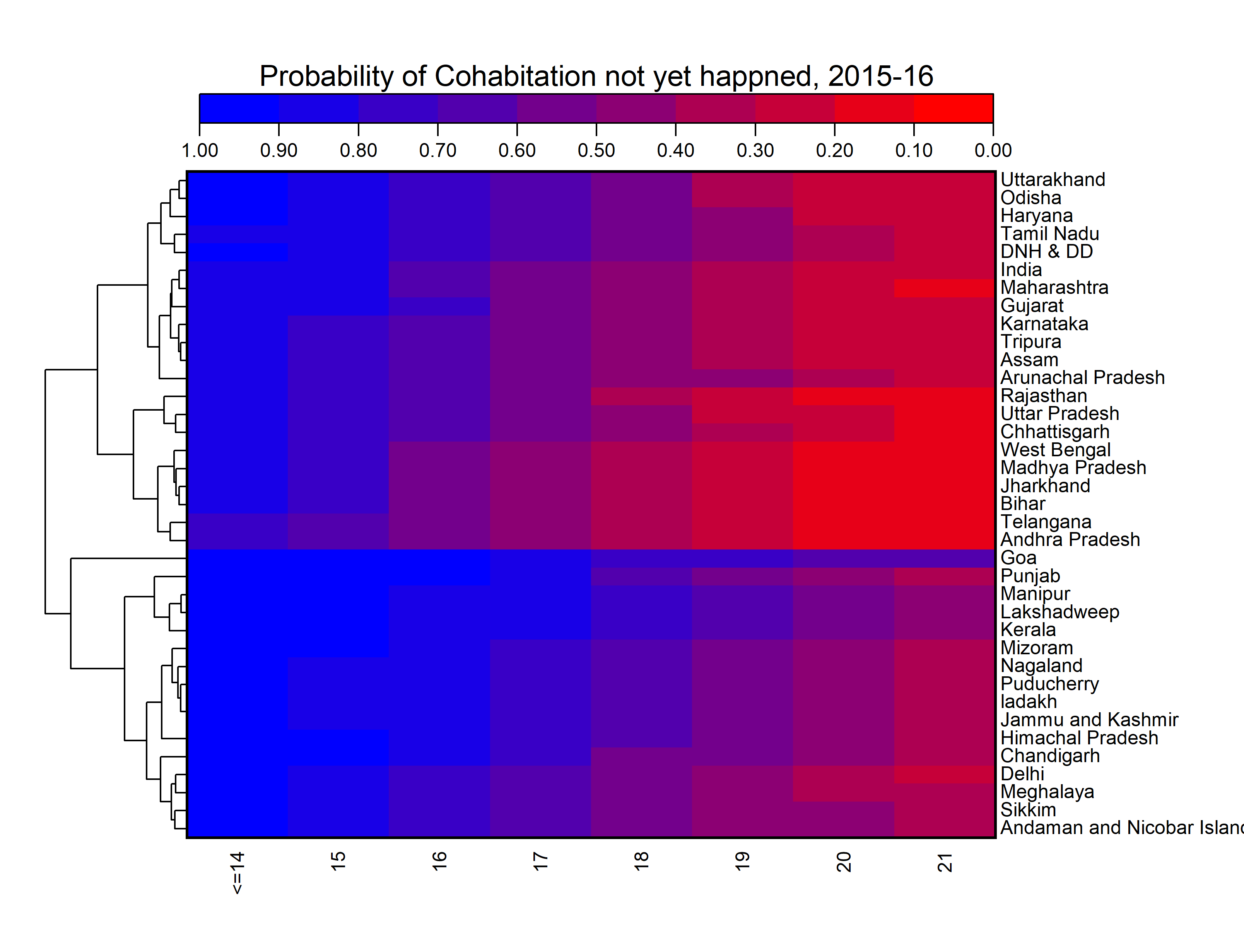 |
| III. 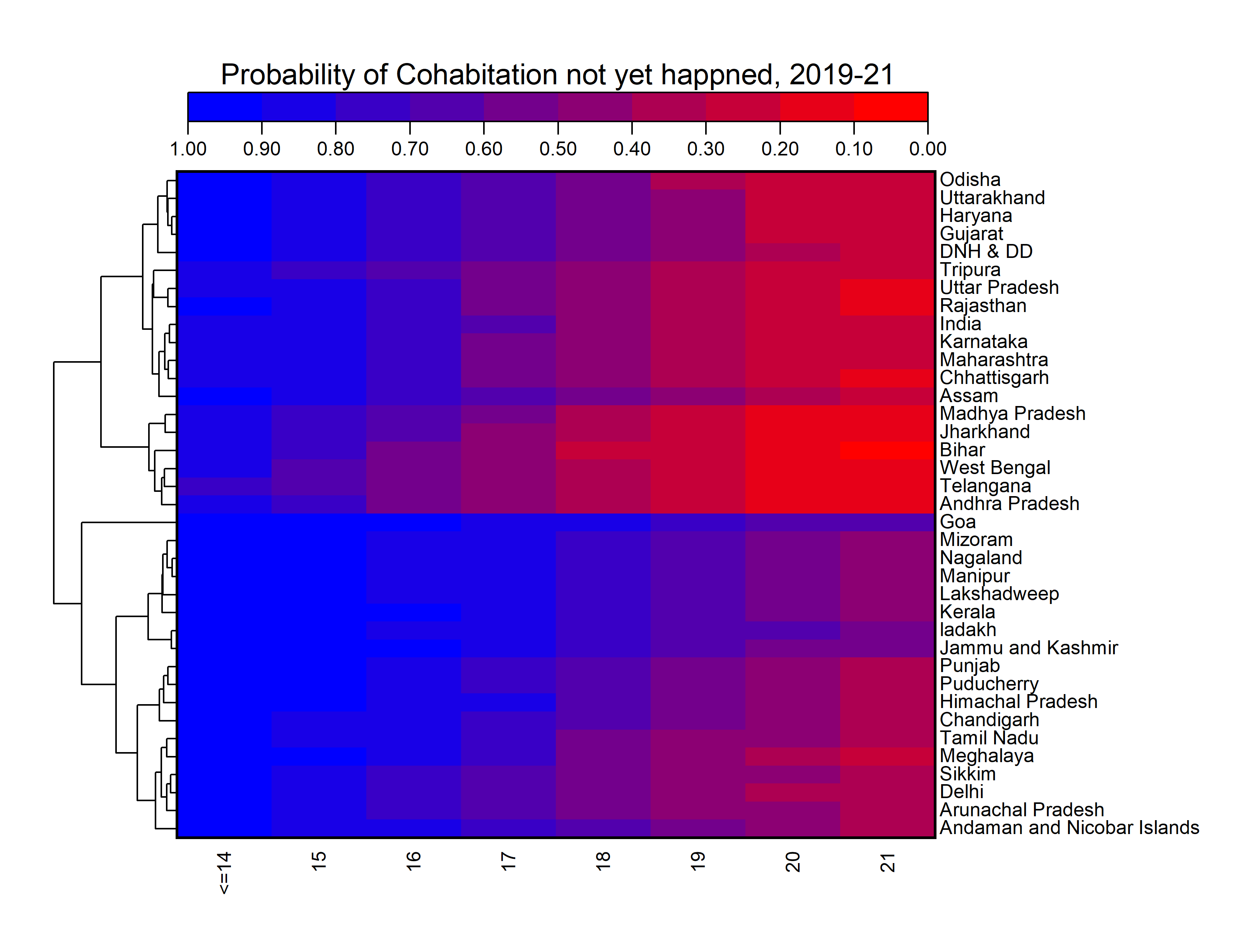 |
| IV. 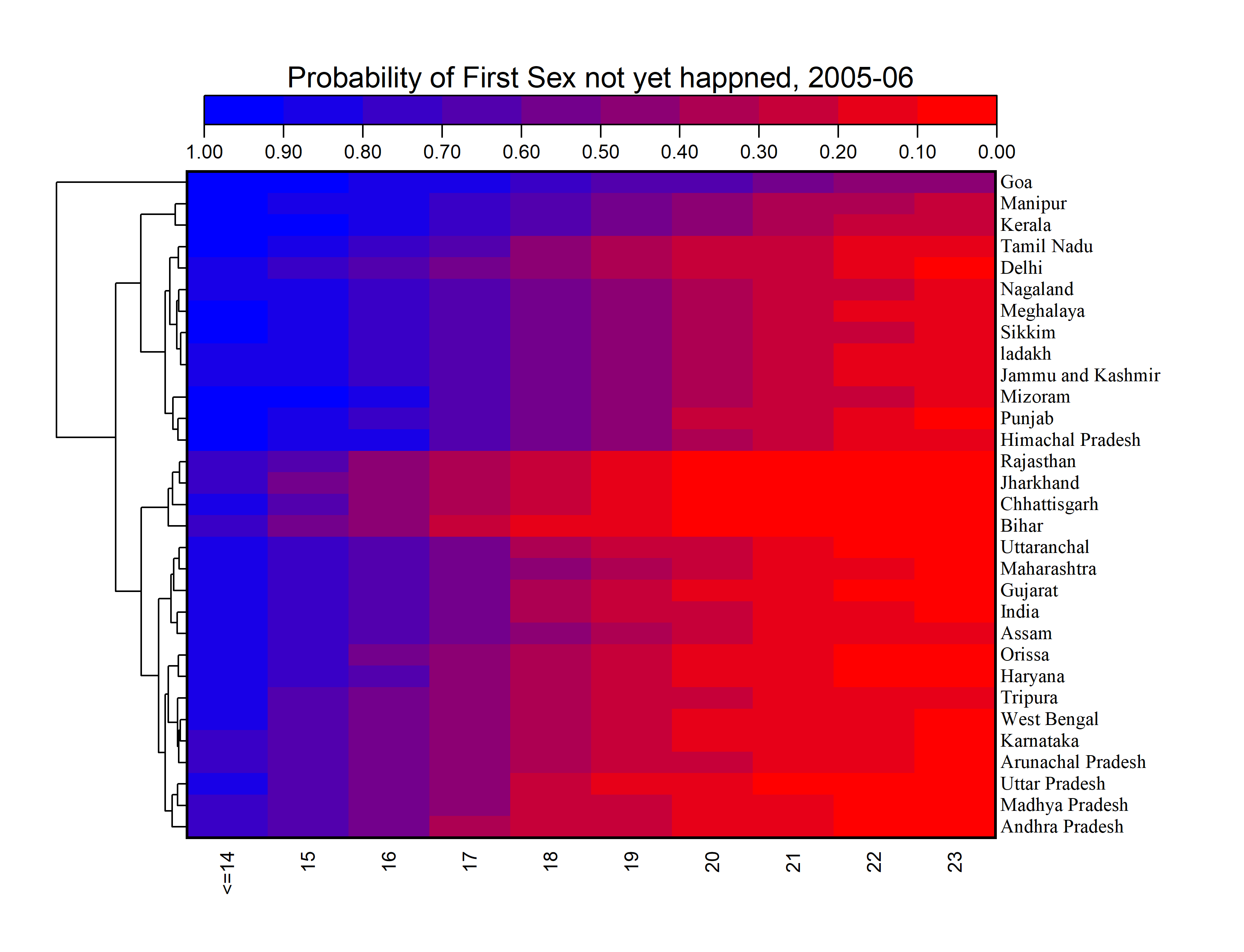 |
| V. 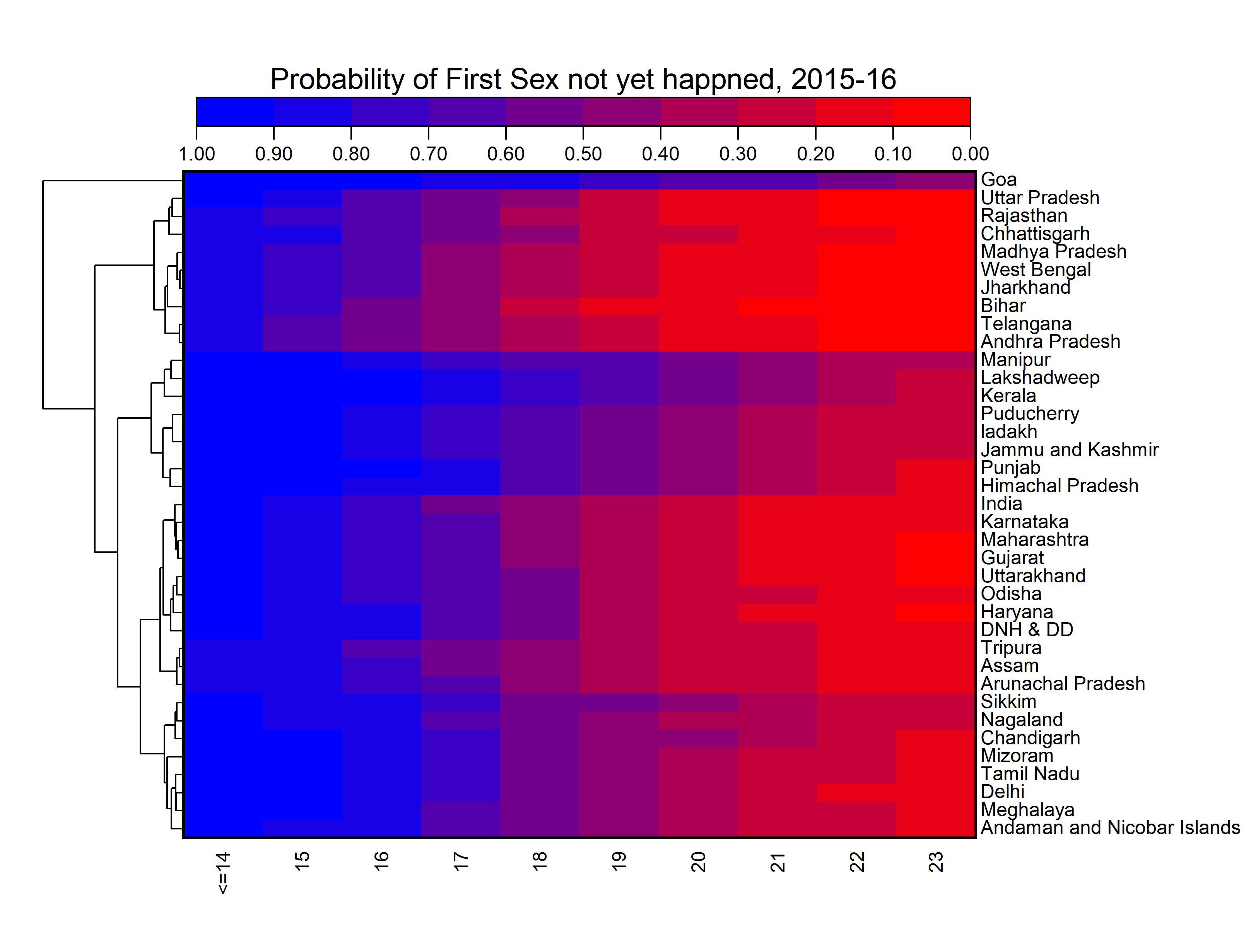 |
| VI. 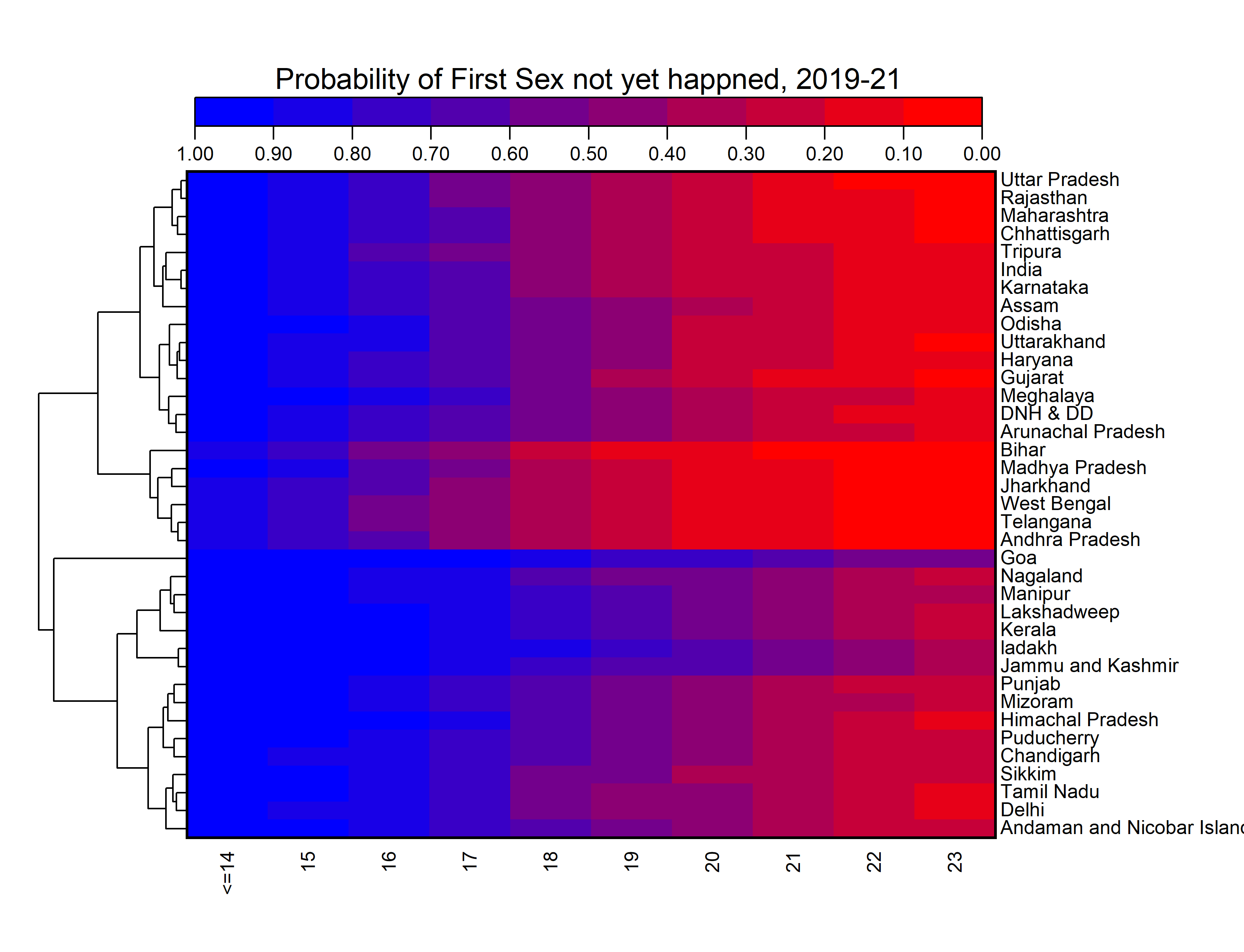 |
| VII. 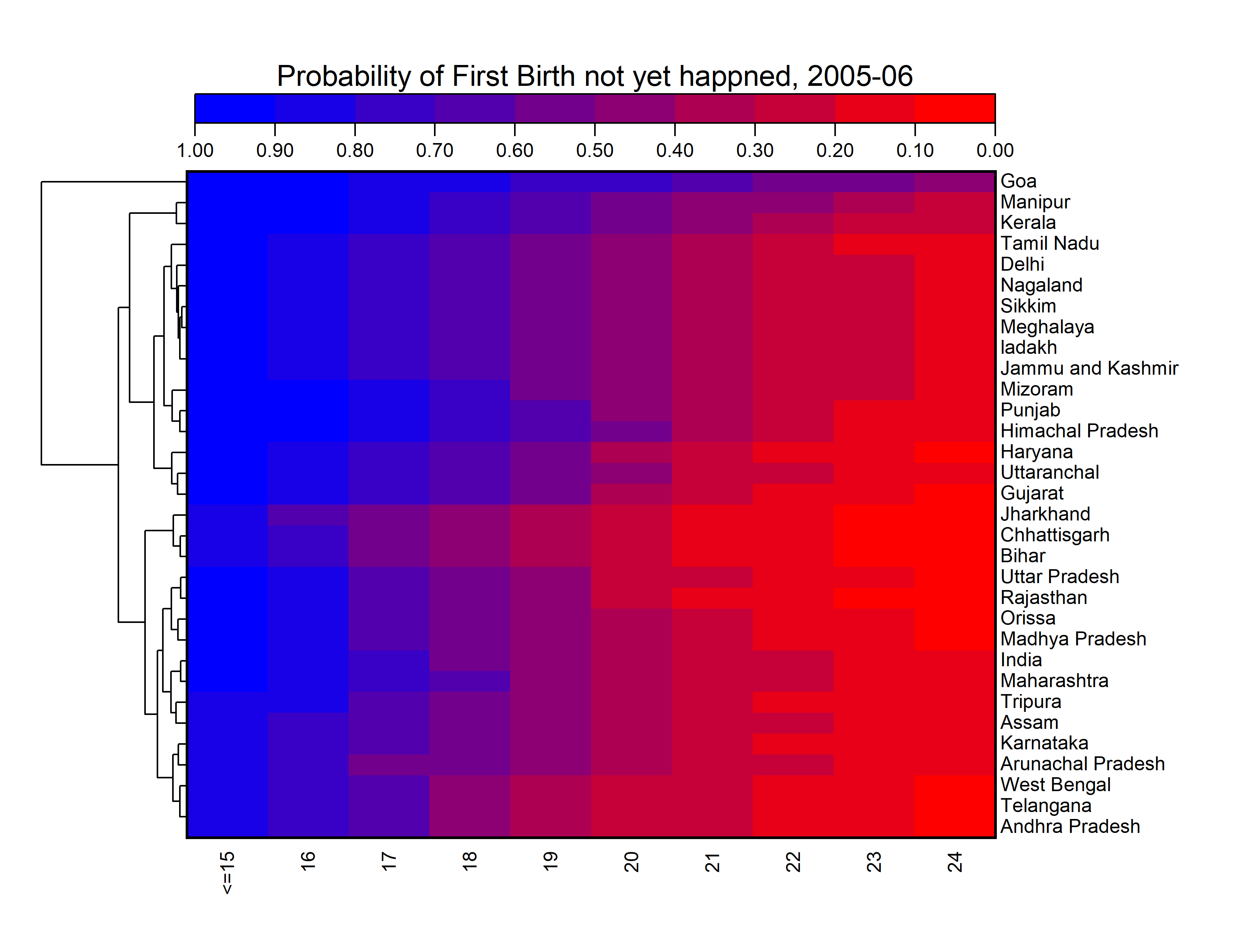 |
| VIII. 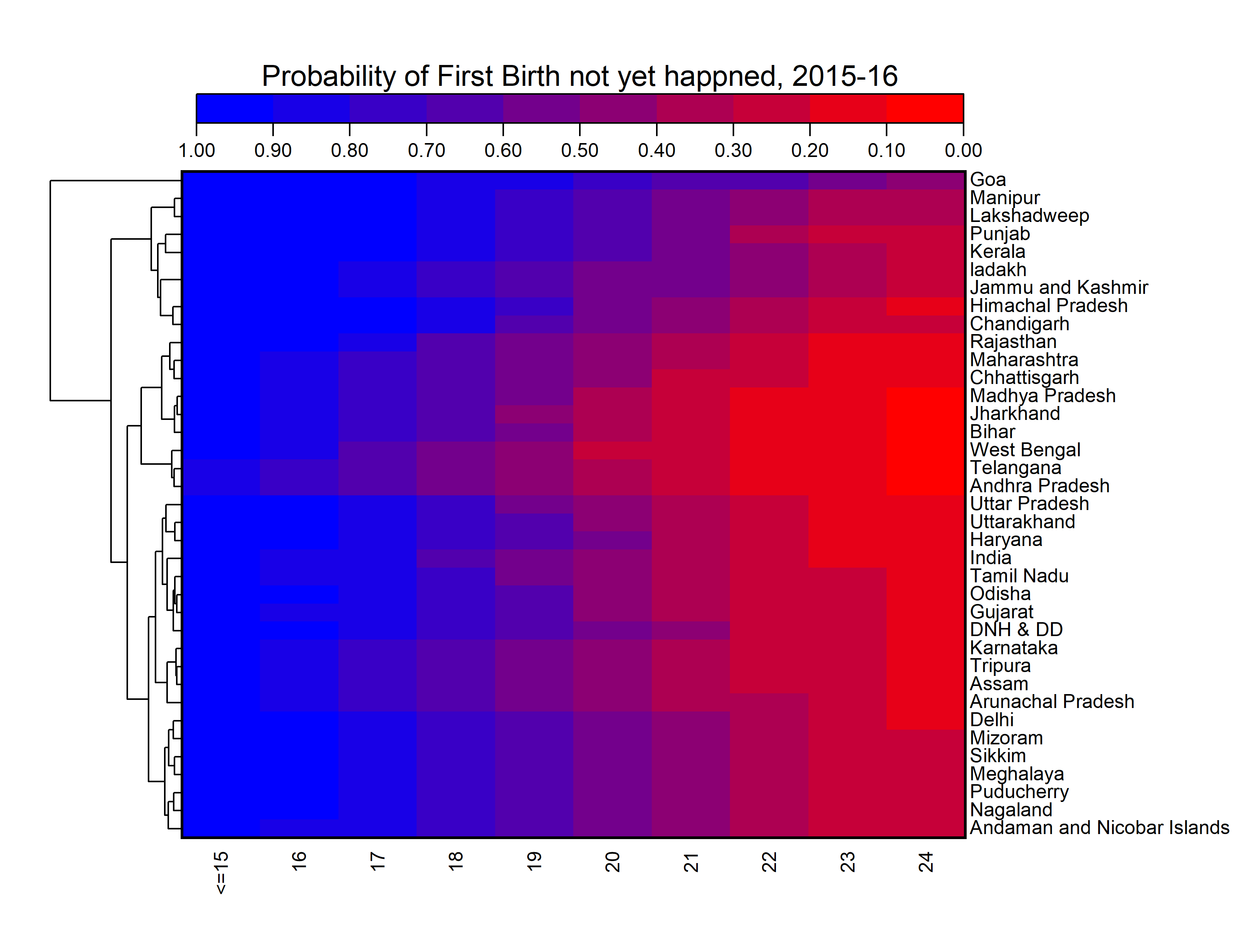 |
| IX. 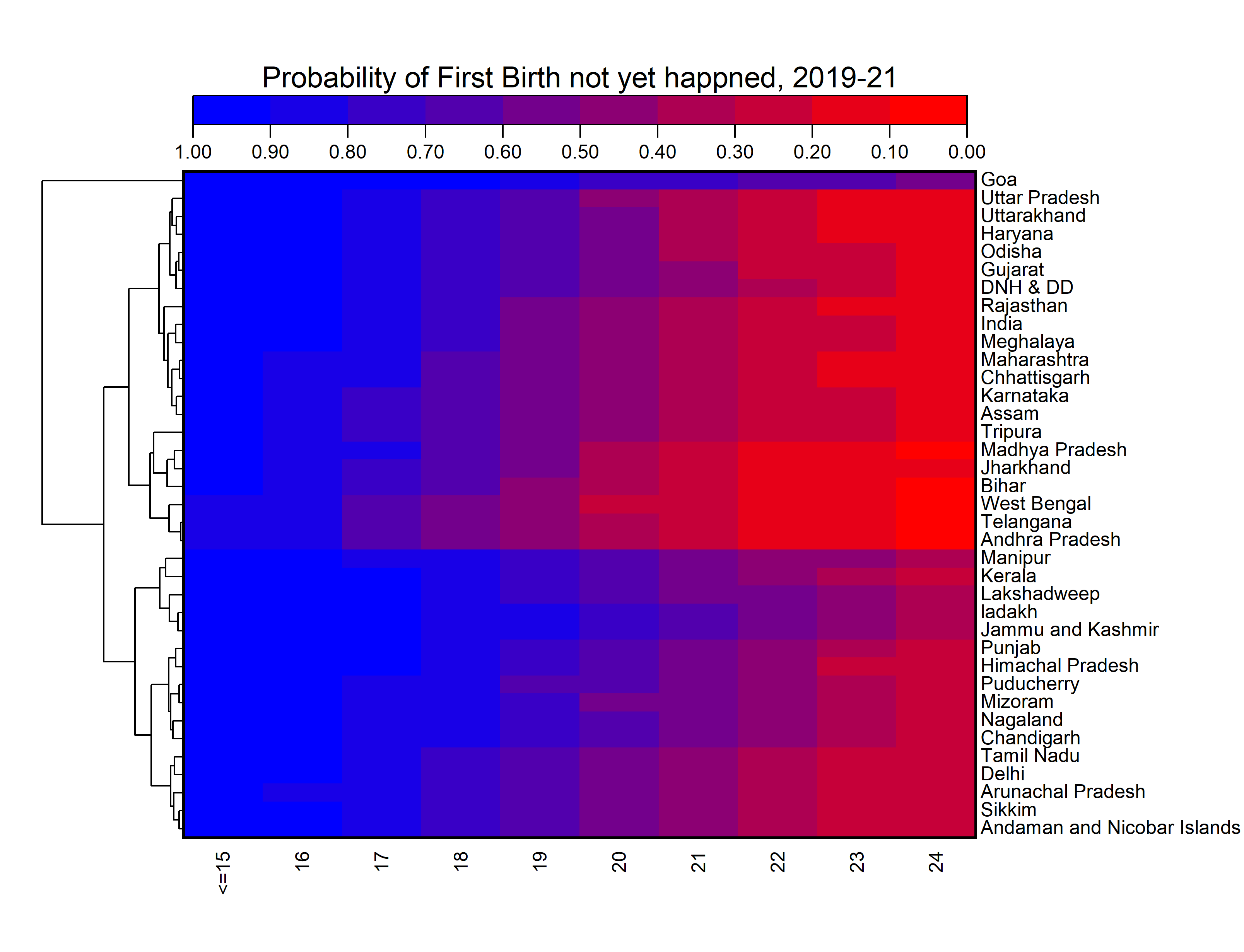 |

**Table B1:** Cox Proportional Hazard Model predicting women risk of first cohabitation, first sex and first birth by various demographic characteristics, Pooled NFHS data 1992-2021.

| **Characteristics** | **First Cohabitation** | | | **First Sex++** | | | **First Birth** | | |
| --- | --- | --- | --- | --- | --- | --- | --- | --- | --- |
| **Individual Characteristics** | **Model 1** | **Model 2** | **Model 3** | **Model 1** | **Model 2** | **Model 3** | **Model 1** | **Model 2** | **Model 3** |
| **Current Age** | **AHR [95% CI]** | | | | | | | | |
| 15-19(Ref) |  |  |  |  |  |  |  |  |  |
| 20-24 | 0.58*** [0.58,0.59] | 0.59*** [0.58,0.59] | 0.61*** [0.60,0.61] | 0.56*** [0.55,0.56] | 0.56*** [0.56,0.57] | 0.57*** [0.56,0.57] | 0.41*** [0.40,0.41] | 0.41*** [0.40,0.41] | 0.42*** [0.42,0.43] |
| 25-29 | 0.45*** [0.45,0.46] | 0.47*** [0.46,0.47] | 0.49*** [0.49,0.50] | 0.43*** [0.43,0.43] | 0.45*** [0.44,0.45] | 0.45*** [0.45,0.46] | 0.28*** [0.27,0.28] | 0.28*** [0.27,0.28] | 0.30*** [0.29,0.30] |
| 30-34 | 0.42*** [0.41,0.42] | 0.44*** [0.43,0.44] | 0.46*** [0.46,0.47] | 0.40*** [0.40,0.40] | 0.43*** [0.42,0.43] | 0.43*** [0.43,0.44] | 0.24*** [0.23,0.24] | 0.24*** [0.24,0.24] | 0.25*** [0.25,0.26] |
| 35-39 | 0.40*** [0.39,0.40] | 0.42*** [0.42,0.42] | 0.45*** [0.45,0.46] | 0.39*** [0.39,0.39] | 0.42*** [0.41,0.42] | 0.43*** [0.42,0.43] | 0.21*** [0.21,0.22] | 0.22*** [0.22,0.22] | 0.23*** [0.23,0.24] |
| 40-44 | 0.40*** [0.39,0.40] | 0.42*** [0.41,0.42] | 0.45*** [0.45,0.46] | 0.39*** [0.39,0.40] | 0.42*** [0.42,0.43] | 0.43*** [0.43,0.44] | 0.21*** [0.20,0.21] | 0.21*** [0.21,0.21] | 0.23*** [0.22,0.23] |
| 45-49 | 0.35*** [0.34,0.35] | 0.37*** [0.36,0.37] | 0.40*** [0.40,0.41] | 0.36*** [0.36,0.37] | 0.39*** [0.39,0.40] | 0.40*** [0.39,0.40] | 0.18*** [0.18,0.18] | 0.18*** [0.18,0.19] | 0.20*** [0.20,0.20] |
| **Education** |  |  |  |  |  |  |  |  |  |
| No Education(Ref) |  |  |  |  |  |  |  |  |  |
| Primary | 0.86*** [0.85,0.86] | 0.87*** [0.87,0.88] | 0.89*** [0.88,0.89] | 0.88*** [0.87,0.88] | 0.89*** [0.88,0.89] | 0.89*** [0.89,0.90] | 0.92*** [0.91,0.92] | 0.91*** [0.91,0.92] | 0.93*** [0.92,0.93] |
| Secondary | 0.60*** [0.59,0.60] | 0.61*** [0.60,0.61] | 0.64*** [0.64,0.65] | 0.61*** [0.60,0.61] | 0.63*** [0.62,0.63] | 0.64*** [0.63,0.64] | 0.67*** [0.66,0.67] | 0.66*** [0.66,0.66] | 0.69*** [0.69,0.70] |
| Higher | 0.35*** [0.34,0.35] | 0.35*** [0.34,0.35] | 0.37*** [0.37,0.37] | 0.35*** [0.34,0.35] | 0.36*** [0.35,0.36] | 0.36*** [0.36,0.37] | 0.39*** [0.38,0.39] | 0.38*** [0.37,0.38] | 0.40*** [0.40,0.40] |
| **Mass Media Exposure** |  |  |  |  |  |  |  |  |  |
| No(Ref) |  |  |  |  |  |  |  |  |  |
| Any | 0.96*** [0.96,0.96] | 0.96*** [0.96,0.97] | 0.99*** [0.98,0.99] | 0.96*** [0.95,0.96] | 0.99*** [0.98,0.99] | 0.98*** [0.98,0.99] | 1.02*** [1.02,1.03] | 1 [1.00,1.01] | 1.03*** [1.02,1.03] |
| **Household Characteristics** |  |  |  |  |  |  |  |  |  |
| **Residence** |  |  |  |  |  |  |  |  |  |
| Urban(Ref) |  |  |  |  |  |  |  |  |  |
| Rural |  | 1.03*** [1.03,1.04] | 1.04*** [1.04,1.04] |  | 1.03*** [1.03,1.04] | 1.04*** [1.04,1.05] |  | 1.02*** [1.01,1.02] | 1.02*** [1.02,1.03] |
| **Caste** |  |  |  |  |  |  |  |  |  |
| SC (Ref) |  |  |  |  |  |  |  |  |  |
| ST |  | 0.91*** [0.90,0.91] | 0.91*** [0.91,0.92] |  | 0.91*** [0.90,0.91] | 0.91*** [0.90,0.92] |  | 0.94*** [0.94,0.95] | 0.95*** [0.94,0.96] |
| Others |  | 0.99*** [0.98,0.99] | 0.97*** [0.96,0.97] |  | 0.98*** [0.97,0.98] | 0.97*** [0.97,0.98] |  | 0.97*** [0.97,0.98] | 0.96*** [0.95,0.96] |
| **Religion** |  |  |  |  |  |  |  |  |  |
| Hindu(Ref) |  |  |  |  |  |  |  |  |  |
| Muslim |  | 0.89*** [0.88,0.89] | 0.91*** [0.90,0.91] |  | 0.88*** [0.87,0.88] | 0.88*** [0.88,0.89] |  | 0.96*** [0.95,0.97] | 0.98*** [0.97,0.98] |
| Christian |  | 0.81*** [0.80,0.81] | 0.79*** [0.79,0.80] |  | 0.86*** [0.86,0.87] | 0.86*** [0.85,0.87] |  | 0.86*** [0.85,0.87] | 0.85*** [0.84,0.86] |
| Others |  | 0.83*** [0.82,0.83] | 0.83*** [0.82,0.83] |  | 0.84*** [0.83,0.85] | 0.84*** [0.83,0.85] |  | 0.88*** [0.87,0.89] | 0.88*** [0.87,0.89] |
| **Wealth Index** |  |  |  |  |  |  |  |  |  |
| Poorest(Ref) |  |  |  |  |  |  |  |  |  |
| Poor |  | 1.07*** [1.06,1.07] | 1.05*** [1.04,1.06] |  | 1.05*** [1.04,1.06] | 1.05*** [1.04,1.05] |  | 1.10*** [1.09,1.11] | 1.08*** [1.08,1.09] |
| Middle |  | 1.07*** [1.07,1.08] | 1.04*** [1.03,1.04] |  | 1.04*** [1.03,1.04] | 1.03*** [1.02,1.04] |  | 1.13*** [1.12,1.14] | 1.09*** [1.09,1.10] |
| Richer |  | 1.04*** [1.04,1.05] | 0.98*** [0.98,0.99] |  | 0.99* [0.98,1.00] | 0.98*** [0.97,0.99] |  | 1.12*** [1.11,1.13] | 1.07*** [1.06,1.07] |
| Richest |  | 1.02*** [1.01,1.03] | 0.93*** [0.92,0.94] |  | 0.95*** [0.94,0.96] | 0.93*** [0.92,0.94] |  | 1.12*** [1.11,1.13] | 1.04*** [1.03,1.05] |
| **State Regions** |  |  |  |  |  |  |  |  |  |
| East(Ref) |  |  |  |  |  |  |  |  |  |
| West |  | 0.90*** [0.90,0.91] | 0.89*** [0.89,0.90] |  | 0.92*** [0.91,0.93] | 0.92*** [0.91,0.92] |  | 0.93*** [0.92,0.94] | 0.92*** [0.91,0.93] |
| North |  | 0.85*** [0.85,0.86] | 0.86*** [0.85,0.86] |  | 0.83*** [0.82,0.83] | 0.83*** [0.83,0.84] |  | 0.86*** [0.86,0.87] | 0.87*** [0.86,0.87] |
| South |  | 0.93*** [0.93,0.94] | 0.92*** [0.92,0.93] |  | 0.90*** [0.89,0.91] | 0.90*** [0.89,0.91] |  | 0.98*** [0.98,0.99] | 0.97*** [0.97,0.98] |
| Central |  | 0.99** [0.99,1.00] | 1.01*** [1.01,1.02] |  | 1.01 [1.00,1.01] | 1.01*** [1.00,1.02] |  | 0.96*** [0.95,0.96] | 0.97*** [0.96,0.97] |
| Northeast |  | 0.75*** [0.74,0.75] | 0.73*** [0.73,0.74] |  | 0.72*** [0.71,0.72] | 0.71*** [0.71,0.72] |  | 0.85*** [0.84,0.86] | 0.84*** [0.83,0.84] |
| **Year of Survey** |  |  |  |  |  |  |  |  |  |
| 1992-93(Ref) |  |  |  |  |  |  |  |  |  |
| 1998-99 |  |  | 1.05*** [1.04,1.06] |  |  |  |  |  | 1.06*** [1.05,1.07] |
| 2005-06 |  |  | 0.96*** [0.95,0.96] |  |  | (Ref) |  |  | 0.95*** [0.94,0.96] |
| 2015-16 |  |  | 0.78*** [0.78,0.79] |  |  | 0.84*** [0.83,0.85] |  |  | 0.79*** [0.79,0.80] |
| 2019-21 |  |  | 0.77*** [0.76,0.78] |  |  | 0.83*** [0.83,0.84] |  |  | 0.80*** [0.79,0.80] |
| **Log Likelihood** | **-17258992** | **-16522981** | **-16517217** | **-14284259** | **-13600719** | **-13599544** | **-15752063** | **-15087735** | **-15083310** |
| **Note:** * p<0.05, ** p<0.01, *** p<0.001, Ref: Reference Category, AHR: Adjusted Hazard Ratio, ++ indicates that for first sex pooling of the data has been done only for the period 2005-06, 2015-16 and 2019-21. | | | | | | | | | |

**Figure A5:** State specific predicted mean age at First Cohabitation, First Sex and First Birth among women aged 15-49 years by survey rounds.

| A1  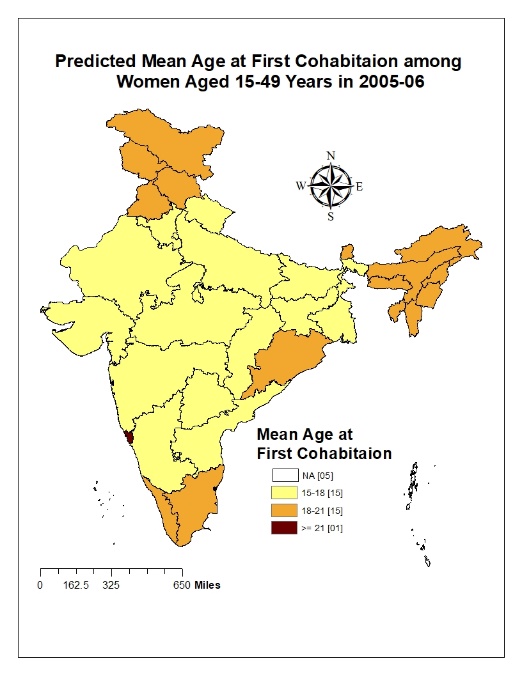 | A2  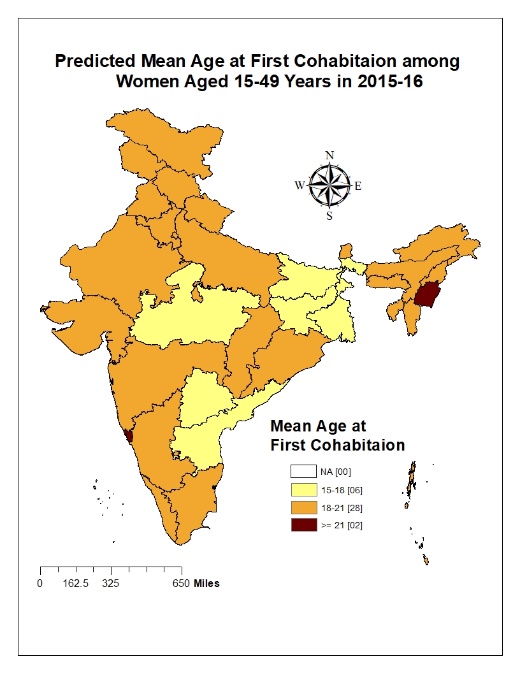 | A3  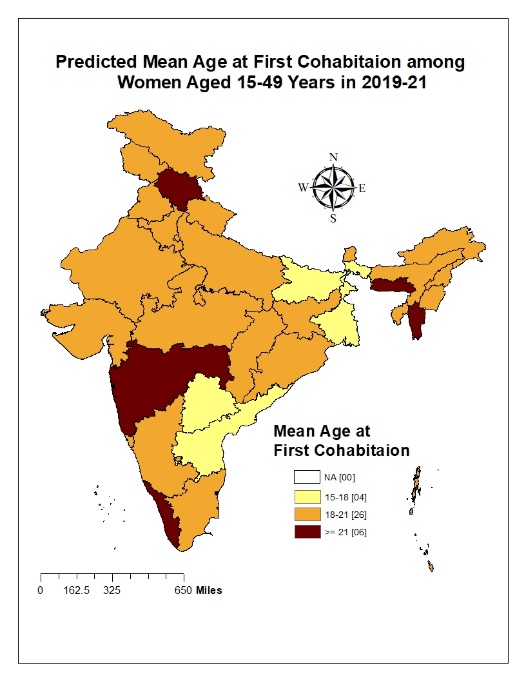 |
| --- | --- | --- |
| B1  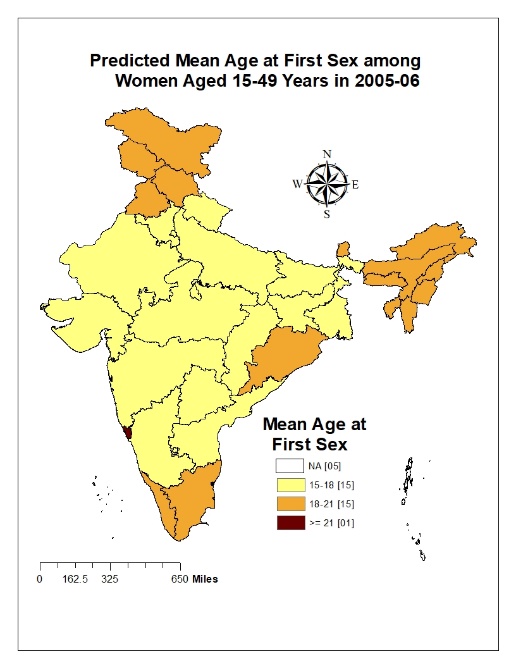 | B2  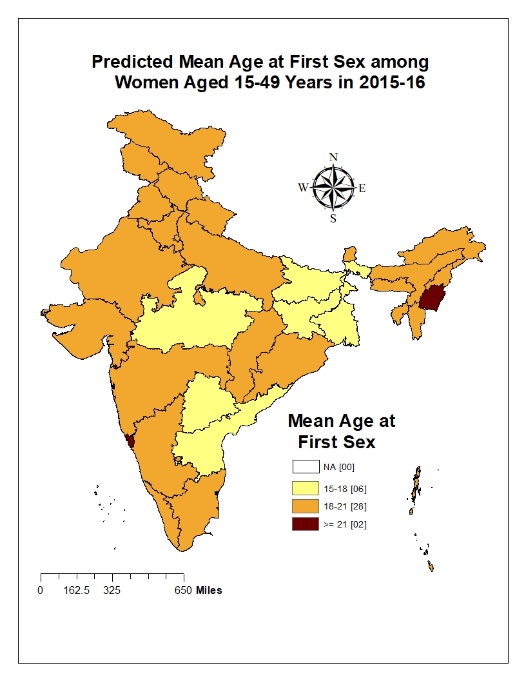 | B3  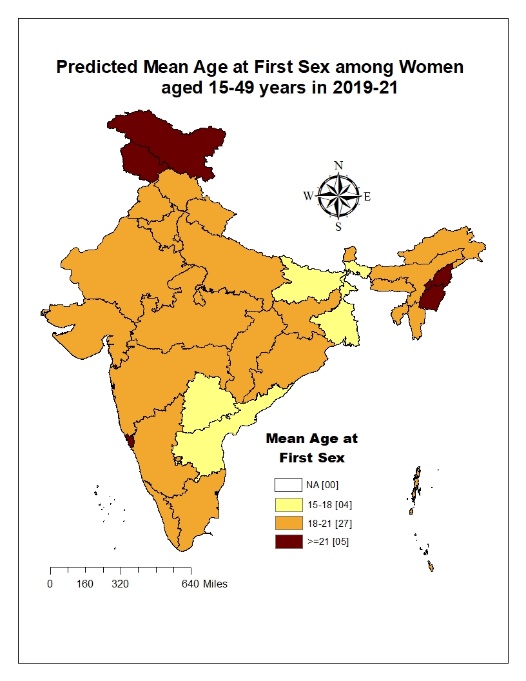 |
| C1  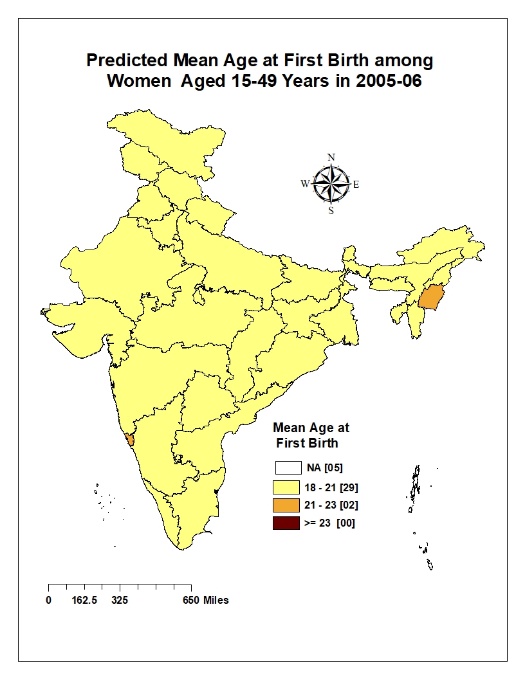 | C2  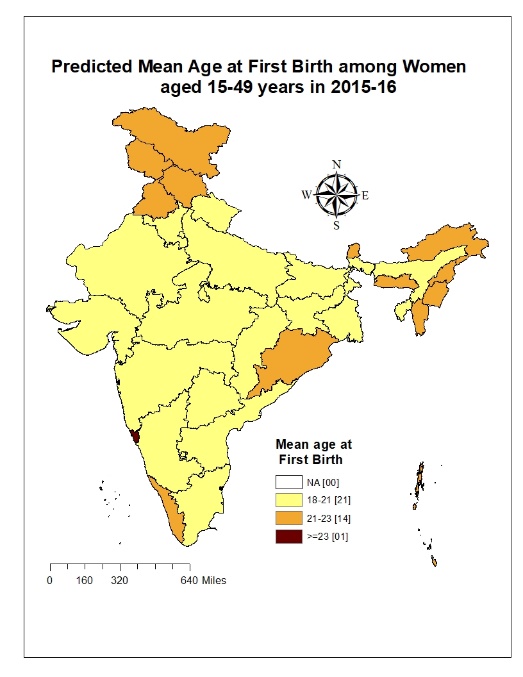 | C3  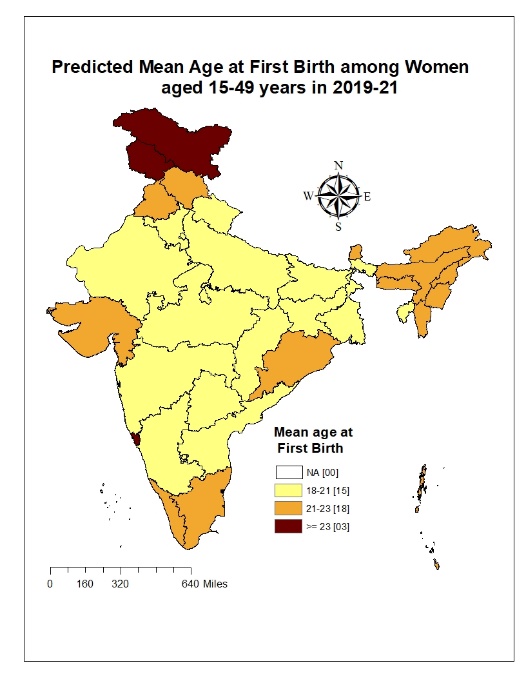 |

**Note:** State specific estimates are adjusted for education, caste, residence, religion and wealth.
